# Solubilities and Transport Properties of CO_2_, Oxalic Acid, and Formic Acid in Mixed Solvents Composed of Deep Eutectic Solvents, Methanol, and Propylene Carbonate

**DOI:** 10.1021/acs.jpcb.2c01425

**Published:** 2022-05-04

**Authors:** Noura Dawass, Jilles Langeveld, Mahinder Ramdin, Elena Pérez-Gallent, Angel A. Villanueva, Erwin J. M. Giling, Jort Langerak, Leo J. P. van den Broeke, Thijs J. H. Vlugt, Othonas A. Moultos

**Affiliations:** † Chemical Engineering Program, Texas A&M University at Qatar, P.O. Box 23874, Doha, Qatar; ‡ Engineering Thermodynamics, Process & Energy Department, Faculty of Mechanical, Maritime and Materials Engineering, Delft University of Technology, Leeghwaterstraat 39, 2628CB Delft, The Netherlands; § Department of Sustainable Process and Energy Systems, TNO, Delft, Zuid-Holland 2628CA, The Netherlands; ∥ Research and Development Department, DMT Environmental Technology, Yndustrywei 3, 8501SN Joure, The Netherlands

## Abstract

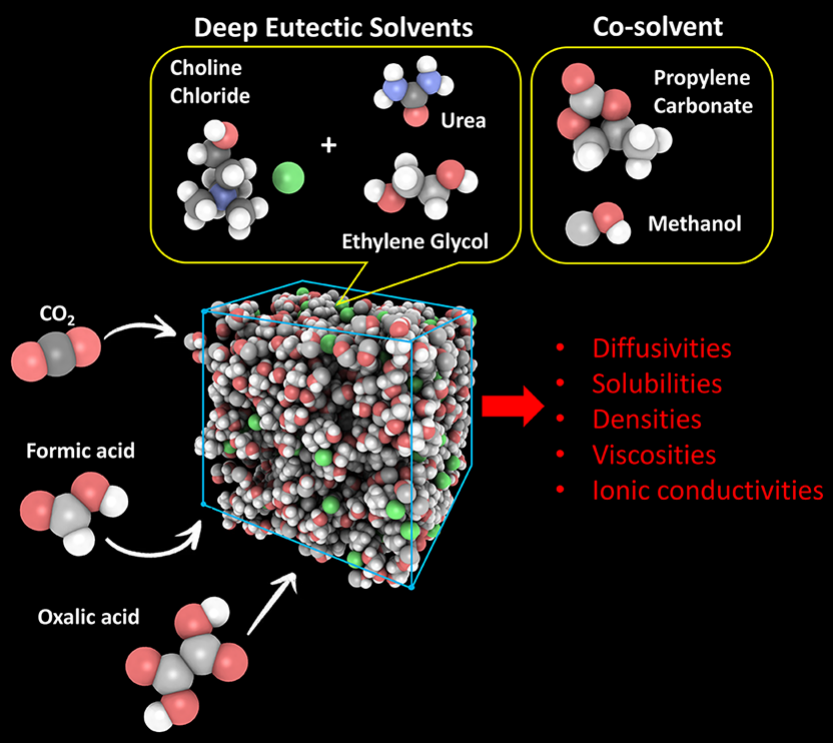

Recently, deep eutectic solvents (DES) have been considered as possible electrolytes for the electrochemical reduction of CO_2_ to value-added products such as formic and oxalic acids. The applicability of pure DES as electrolytes is hindered by high viscosities. Mixtures of DES with organic solvents can be a promising way of designing superior electrolytes by exploiting the advantages of each solvent type. In this study, densities, viscosities, diffusivities, and ionic conductivities of mixed solvents comprising DES (i.e., reline and ethaline), methanol, and propylene carbonate were computed using molecular simulations. To provide a quantitative assessment of the affinity and mass transport of CO_2_ and oxalic and formic acids in the mixed solvents, the solubilities and self-diffusivities of these solutes were also computed. Our results show that the addition of DES to the organic solvents enhances the solubilities of oxalic and formic acids, while the solubility of CO_2_ in the ethaline-containing mixtures are in the same order of magnitude with the respective pure organic components. A monotonic increase in the densities and viscosities of the mixed solvents is observed as the mole fraction of DES in the mixture increases, with the exception of the density of ethaline-propylene carbonate which shows the opposite behavior due to the high viscosity of the pure organic component. The self-diffusivities of all species in the mixtures significantly decrease as the mole fraction of DES approaches unity. Similarly, the self-diffusivities of the dissolved CO_2_ and the oxalic and formic acids also decrease by at least 1 order of magnitude as the composition of the mixture shifts from the pure organic component to pure DES. The computed ionic conductivities of all mixed solvents show a maximum value for mole fractions of DES in the range from 0.2 to 0.6 and decrease as more DES is added to the mixtures. Since for most mixtures studied here no prior experimental measurements exist, our findings can serve as a first data set based on which further investigation of DES-containing electrolyte solutions can be performed for the electrochemical reduction of CO_2_ to useful chemicals.

## 1 Introduction

During the past few decades, carbon capture, utilization, and storage (CCUS) technologies have been in the spotlight of the academic and industrial research as a means for reducing the concentration of CO_2_ in the atmosphere.^1^ One promising CCUS route is the utilization (e.g., reduction) of CO_2_ as a feedstock for the production of value-added products.^2,3^ Several technologies are available for the reduction of CO_2_, e.g., photocatalytic, thermal, and electrochemical. Electrochemical processes have distinct advantages such as the lack of complex reaction pathways, cost-efficiency, and relatively high reduction efficiencies.^4−6^ CO_2_ can be electrochemically converted to a number of valuable materials and fuels, spanning polymers, acids, alcohols, and hydrocarbons.^3,7^ Valuable CO_2_ electroreduction products include formic and oxalic acid, which are the simplest forms of monocarboxylic and dicarboxylic acids, respectively.^8,9^ The CO_2_ electoreduction to these acids require only two moles of electrons per mole of product and have a high market price.^7,10^ Formic acid can be produced with high Faraday efficiencies (>95%) and current densities (150 mA cm^–2^) using gas diffusion electrodes.^10^ In 2018, formic acid was reported to have a total market value of $756.5 MM, with a market price of approximately $400/tonne. Formic acid is mostly used in agriculture, the production of leather and textiles, and in the pharmaceutical industry.^11^ Oxalic acid is mainly used in the pharmaceutical and textile industry.^12,13^ Oxalic acid has a global market value of $715 MM and a market price of ca. $500/tonne.^14^

Despite the tremendous progress that has been made in the field of electrochemical processes during the past few decades, significant challenges and limitations still remain.^6^ The main challenges are the high overpotential requirements and the low selectivity toward the desired products. To overcome these limitations, many factors have to be considered when designing and optimizing an electrochemical conversion process, e.g., the electrochemical cell configuration, catalyst, and type of electrolyte.^7,15^ The role of the electrolyte is of particular importance since it constitutes the medium for the conversion reactions and controls the transport of the different chemical species to the catalysts.^15^ Consequently, selecting the optimum electrolyte/solvent for a conversion process can enhance the performance of electrochemical conversion processes.^16^ To this purpose, many electrolytes have been tested through the years, e.g., aqueous and organic solvents and ionic liquids (ILs).^5^ ILs have been considered for these processes due to high thermal stability, ionic conductivity, and absorption of CO_2_. The use of ILs has also been shown to reduce the required overpotential and undesirable side reactions in electrochemical conversions, while the ILs themselves can act as a co-catalyst.^17,18^

Deep Eutectic Solvents (DES) are an emerging class of solvents sharing similar properties and advantages with ILs.^19−28^ Many DES, e.g., choline-based, can be easily prepared from mixing naturally occurring substances and, thus, are cheaper to produce than most ILs.^29,30^ Compared to ILs, the use of DES in electrochemical processes is not as widely investigated. High viscosities can be a limiting factor toward application of pure DES as electrolytes for the electrochemical reduction of CO_2_.^31^ To exploit the benefits of DES in such processes while overcoming the drawbacks, mixing DES with other solvents has been considered. Vasilyev et al.^31^ showed that the CO_2_ reduction reaction takes place in the presence of various choline-based DES, such as reline and ethaline (which are formed by mixing choline chloride with urea and ethylene glycol, respectively, in the ratio 1:2). Vasilyev et al.^31^ also observed that the efficiency of CO_2_ reduction increased upon the addition of DES in the originally used electrolyte, i.e., ethylene glycol.

A first approach for examining the feasibility of solvents containing DES in electrochemical applications is to investigate the thermo-physical properties of these solvents and of the respective mixtures with the reactants and products. For example, the solubility and diffusivity of solutes (e.g., CO_2_, products) in electrolytes are very important properties since they often are limiting factors in electrochemical conversions. Excess properties and solubilities of the solutes in the solvents are equally important for, e.g., the design of downstream separation processes following the conversion of CO_2_ to the value-added products. While experiments are traditionally used to measure properties of fluid mixtures, molecular simulations are less costly and, therefore, can assist in the initial screening of a large number of solvents for electrochemical processes. Molecular simulation also provides the necessary fundamental understanding of the physical/chemical mechanisms at the atomistic scale. For these reasons, molecular simulations have been widely used to compute various properties relevant to electrochemical applications.^32−40^

In this work, the solubilities and self-diffusivities of CO_2_, oxalic acid, and formic acid in mixtures of DES with organic solvents are computed by means of Monte Carlo (MC) and Molecular Dynamics (MD) simulations. Self-diffusivities, densities, viscosities, and ionic conductivities of the solvent mixtures are also computed as a function of the composition of the mixtures. Two DES, i.e., reline and ethaline, are considered here. The organic solvents considered are methanol and proplyene carbonate. These solvents have been used as electrolytes for the conversion of CO_2_ to formic acid and oxalic acid, respectively.^41-44^ Our study shows that the reline–methanol mixtures have slightly lower affinity toward CO_2_ and that the addition of DES to the organic solvents increase the solubilities of oxalic and formic acids. The densities and viscosities increase with the mole fraction of DES, except for the density of ethylene-propylene carbonate (due to the higher density of the pure organic component compared to the DES). In contrast, the self-diffusivities of all molecular species vastly decrease due to the increasing viscosity. For all mixed solvents, the ionic conductivities show a nonmonotonic behavior with the DES content. Initially, the ionic conductivity increases until a maximum value, and then a sharp decrease is observed as more DES is added. This behavior is in line with prior studies on aqueous DES solutions, and reline–ethaline mixtures.^26,45,46^ Overall, comparisons of our simulation data with the limited available experimental measurements are in reasonable agreement.

This paper is organized as follows. In Section 2, the computational details regarding the MC and MD simulations and the force fields used are provided. The results of the thermodynamic and transport properties are presented in Section 3. In the same section, an analysis of the hydrogen bonding behavior of the system is performed. The conclusions of this study are discussed in Section 4.

## 2 Methods

Molecular simulations are performed for the following solvents: methanol, propylene carbonate, reline, ethaline, and mixtures of ethaline-propylene carbonate, ethaline-methanol, and reline-methanol. The mole fraction of DES in the different mixtures is defined as follows:
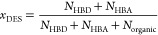
1where *N*_HBD_, *N*_HBA_, and *N*_organic_ is the number of hydrogen bond donors, acceptors, and organic molecules. For example, in the case of ethaline-methanol mixtures, *N*_HBD_, *N*_HBA_, and *N*_organic_ correspond to the total number of ethylene glycol, choline chloride, and methanol molecules, respectively.

### 2.1 Force Fields

Nonpolarizable force fields consisting of bonded (i.e., bond streching, angle bending, and torsions) and nonbonded (i.e., Lennard-Jones and Coulombic) terms were used to simulate all species in this work. The TraPPE force field was used to model CO_2_^47^ and methanol.^48^ For oxalic acid, the modified OPLS force field proposed by Doherty and co-workers^49,50^ was used. Formic acid was modeled using the modified OPLS force field parametrized by Salas et al.^49,51^ which yields improved predictions for the dielectric constant. Propylene carbonate parameters were taken from the work of Silva and Freitas,^52^ who adopted GAFF and refitted the charges. The DES were modeled using the GAFF^53^ force field consistently with our previous studies.^21,24,26,27,54^ For choline, urea, and ethylene glycol, 1–4 interactions were scaled by a factor of 0.5 for both Lennard-Jones and Coulombic interactions. The charges of choline chloride were scaled by a factor of 0.8 and 0.9 in reline and ethaline, respectively.^55,56^ This implementation yields accurate predictions for various thermophysical properties of DES as shown by Perkins et al.,^55,56^ Salehi et al.,^24^ and Celebi et al.^26,27,57^ The Lennard-Jones interaction parameters between unlike species were computed using the Lorentz–Berthelot combining rules.^58^ All force field parameters and the functional forms of the bonded and nonbonded terms used in this study are available in the Supporting Information.

### 2.2 Monte Carlo Simulations

In this work, MC simulations were performed to compute the excess chemical potentials (μ^ex^) and Henry coefficients (*H*), which are used to quantify the solubilities of solutes (i.e., CO_2_, oxalic acid, and formic acid) in different mixed solvents. For a component *i*, the excess chemical potential μ_*i*_^ex^ follows from^59^
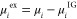
2where μ_*i*_ and μ_*i*_^IG^ are the chemical potentials of the component and the ideal gas at the same conditions, respectively. For a specific solute–solvent combination, μ_*i*_^ex^ indicates the affinity of the solute toward the solvent as it is related to the activity coefficient γ_*i*_ of component *i*:^60,61^
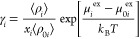
3where ⟨ρ_0*i*_⟩ is the ensemble average number density of pure component *i*, ⟨ρ_*i*_⟩ is the ensemble average number density of *i*, *x*_*i*_ is the mole fraction of *i*, μ_0*i*_^ex^ is the excess chemical potential of pure *i* with respect to the ideal gas, *k*_B_ is the Boltzmann constant, and *T* is the temperature in units of K. The Henry coefficient of the solute, *H*_*i*_ is defined as^59^
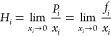
4where *P*_*i*_ and *f*_*i*_ are the partial pressure and fugacity of the solute, respectively. *H*_*i*_ is directly related to μ_*i*_^ex^ as follows:^62^
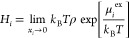
5where ρ is the number density of the solvent and *T* is the temperature in units of K.

All MC simulations were carried out with the open-source software package Brick-CFCMC,^63,64^ which utilizes the Continuous Fractional Component (CFC) method^65,66^ (i.e., gradual insertion/deletion of fractional molecules during the simulations). The degree of interaction between a fractional with the surrounding molecules is varied using a scaling parameter λ (0≤ λ ≤ 1), which is a degree of freedom in an expanded ensemble formulation.^67^ For more details on the CFCMC method, the reader is referred elsewhere.^65−69^ Recently, a thermodynamic integration feature has been developed in Brick-CFCMC for computing μ^ex^ based on^64^
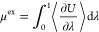
6where *U* is the energy of the system, and the brackets ⟨···⟩ denote an ensemble average. During CFCMC simulations, separate scaling parameters are used for intermolecular Lennard-Jones and electrostatic interactions. The scaling parameters are continuous functions of λ and are implemented such that electrostatic interactions are not switched on before fully scaling down the Lennard-Jones interactions. For more details, including the scaling functions, the reader is referred to the work of Polat et al.^64^ To compute μ^ex^ of CO_2_, oxalic acid, and formic acid in different solvents using eq 6, the λ space was discretized into 50 bins. Separate simulations in the *NPT* ensemble were performed for each solute with a fixed value of λ to compute . Subsequently, numerical integration of eq 6 was performed. More details on the thermodynamic integration feature of Brick-CFCMC can be found in the recent work of Polat et al.^64^ μ^ex^ and *H* were computed for mixtures with 0 ≤ *x*_DES_ ≤ 0.4 at 298.15 K and 1 atm and for pure reline and ethaline at 350.15 K and 1 atm.

A cutoff radius of 12 Å was used for both the Lennard-Jones and the Coulombic potential in all MC simulations except for the ones of pure DES in which a cutoff radius of 10 Å was used. Electrostatic interactions were handled with the Ewald summation method with a relative precision of 10^-6^. During the MC simulations, trial moves were selected with the following probabilities: 35% translations, 35% rotations, 29% changes in the internal configuration of molecules (i.e., angles and dihedrals), and 1% volume changes. A minimum of 8 × 10^5^ cycles were carried out for equilibration and 8 × 10^5^ cycles for production. At each MC cycle, the number of the trial moves performed equals the number of molecules of the system.

### 2.3 Molecular Dynamics Simulations

MD simulations were performed for the computation of the densities, number of hydrogen bonds (HBs), shear viscosities, and self-diffusion coefficients. All MD simulations were carried out using the large-scale atomic/molecular massively parallel simulator (LAMMPS).^70^ The initial configurations were generated with the PACKMOL package.^71^ Long-range electrostatic interactions were handled using the particle–particle particle-mesh (PPPM) method with a relative error of 10^–6^. The cutoff radius was set to 12 Å for both Lennard-Jones and the short-range part of the Coulombic interactions. Periodic boundary conditions were imposed in all directions. The Verlet algorithm with a time step of 1 fs was used to integrate Newton’s equations of motion. Temperature and pressure were maintained constant using the Nose–Hoover thermostat and barostat with coupling constants of 100 and 1000 fs, respectively.

Transport properties were computed with the OCTP (on-the-fly computation of transport properties) plugin in LAMMPS^72^ which yields the mean-squared displacements (MSDs) of dynamical properties as a function of time. The transport coefficients can be then obtained by linear regression to the long-time MSDs at time-scales where the slopes as a function of time are equal to 1 in a log–log plot. Diffusion coefficients are computed from^58,72^
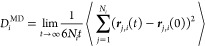
7where *D*_*i*_^MD^ is the self-diffusivity of species *i*, ***r***_*j*,*i*_(*t*) is the position vector of the *j*^th^ molecule of species *i* at time *t*, and *N*_*i*_ is the number of molecules of species *i* in the system. The shear viscosity η follows from^58,72^
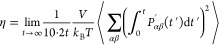
8where^73^
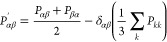
9where *V* is the volume of the system, *P*_*αβ*_^′^ are the components of the traceless pressure tensor, *P*_*αβ*_ are the off-diagonal components of the pressure tensor, and δ_*αβ*_ is the Kronecker delta. All self-diffusion coefficients were corrected for finite-size effects using the Yeh-Hummer (YH) equation:^74−76^
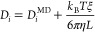
10where *D*_*i*_ is the corrected self-diffusion coefficient corresponding to the thermodynamic limit, η is computed from MD simulations and does not depend on the system size,^77,78^ and ξ is a dimensionless constant equal to 2.837298 for a periodic cubic simulation box. To compute the ionic conductivities, the Nernst–Einstein (NE) equation was used:^79^
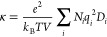
11where *e* is the elementary charge and *q*_*i*_ is the charge of the molecules of species *i*. Eq 11 has been shown to be a relatively good approximation for obtaining the ionic conductivities of ionic species in a computationally efficient way.^26,54,80,81^ For all mixtures considered here, only the charges and the self-diffusivities of choline and chloride were used in the NE equation since the rest of the species are charge-neutral (i.e., HBDs and the organic solvents). The ionic conductivity can also be computed using the appropriate Green–Kubo and Einstein relations (i.e., cross correlation of charge fluxes/displacements).^79^

The MD simulations of the solvents with *x*_DES_ ranging from 0 to 1 were performed at 298.15 K and 1 atm. A list of the solvents studied here and the number of molecules used for each species is shown in Table 1. For the computation of the self-diffusivities of CO_2_, oxalic acid, and formic acid in the different solvents, five solute molecules were used. This helps to drastically improve the sampling of MSDs, while it practically corresponds to infinite dilution. The MD simulation scheme was as follows. Initially, an energy minimization using the conjugate-gradient method with a tolerance of 10^–4^ was performed. Then, equilibration runs in the *NPT* ensemble were carried out for 10–20 ns, depending on *x*_DES_. Finally, production runs of 10–100 ns were carried out in the *NVT* ensemble from which all properties were computed. For each system, averages and standard deviations were computed over 5 independent MD simulations, each one starting from a different initial configuration. Visual molecular dynamics (VMD)^82^ was used for the HB analysis. The criterion for the formation of a HB was a cutoff distance of 3.5 Å between the donor and acceptor atoms and a cutoff angle of 30° between the donor-hydrogen-acceptor atoms.^83,84^

**Table 1:** Number of Molecules Used in the MD Simulations for Every Solvent: Choline (Ch^+^), Chloride (Cl^–^), Ethylene Glycol (EG), Methanol (MeOH), and Propylene Carbonate (PC)^a^

solvent	*x*_DES_	Ch^+^	Cl^–^	urea	EG	MeOH	PC
reline-MeOH	0	–	–	–	–	800	–
reline-MeOH	0.1	100	100	200	–	3600	–
reline-MeOH	0.2	100	100	200	–	1600	–
reline-MeOH	0.4	100	100	200	–	600	–
reline-MeOH	0.6	125	125	250	–	333	–
reline-MeOH	0.8	150	150	300	–	150	–
reline-MeOH	1	00	200	400	–		–
ethaline-MeOH	0			–		800	–
ethaline-MeOH	0.1	100	100	–	200	3600	–
ethaline-MeOH	0.2	100	100	–	200	1600	–
ethaline-MeOH	0.4	100	100	–	200	600	–
ethaline-MeOH	0.6	125	125	–	300	333	–
ethaline-MeOH	0.8	150	150	–	300	150	–
ethaline-MeOH	1	200	200	–	400	–	–
ethaline-PC	0			–		–	400
ethaline-PC	0.1	25	25	–	50	–	900
ethaline-PC	0.2	50	50	–	100	–	800
ethaline-PC	0.4	75	75	–	150	–	450
ethaline-PC	0.6	125	125	–	250	–	333
ethaline-PC	0.8	150	150	–	300	–	150
ethaline-PC	1	200	200	–	400	–	–

a For the computation of self-diffusivities of CO_2_, formic acid, and oxalic acid in these solvents, five solute molecules were used for each case.

## 3 Results and Discussion

### 3.1 Thermodynamic Properties

#### 3.1.1 Densities

**Figure 1:**
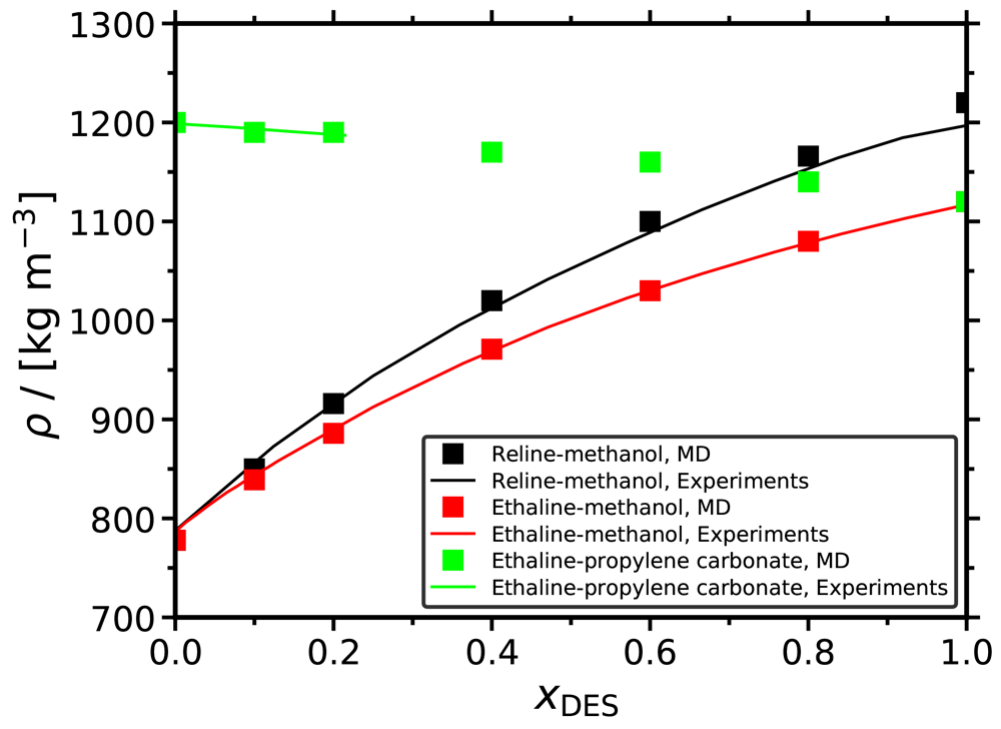
Densities of the ethaline-propylene carbonate, ethaline-methanol, and reline-methanol mixtures as a function of the mole fraction of DES at 298 K and 1 atm. The black, red, and green lines represent experimental measurements by Haghbakhsh et al.^94^ (reline-methanol), Wang et al.^86^ (ethaline-methanol), and Zafarani-Moattar et al.^87^ (ethaline-propylene carbonate), respectively. The error bars of the MD data are smaller than the symbol size. Tabulated values of the computed densities are presented in the Supporting Information.

Figure 1 shows a comparison between the densities computed in MD simulations and the available experimental measurements for the DES-organic solvent mixtures as a function of *x*_DES_. The MD results are in excellent agreement with the experiments for all systems with maximum absolute deviations of 1.2%, 1.1%, and 1.0% for reline-methanol, ethaline-methanol, and ethaline-propylene carbonate mixtures, respectively. These low deviations serve as validation of the accuracy of the selected force fields.

As expected, the densities of the methanol-containing solvents increase considerably with the addition of DES, due to the large difference between the densities of the pure components (i.e., the densities of methanol, ethaline, and reline are 778, 1120, and 1200 kg/m^3^, respectively). Reline-methanol mixtures are denser than ethaline-methanol mixtures for any *x*_DES_. This is also expected since the density of pure reline is higher than that of pure ethaline. Ethaline-propylene carbonate mixtures have higher densities compared to the methanol-containing ones for *x*_DES_ ≤ 0.8. In these systems, the density decreases with the addition of DES (opposite behavior from the methanol mixtures); however, this decrease is not large. The density of ethaline–propylene carbonate mixtures decrease by 5% as *x*_DES_ increases from 0 to 0.8. This is mainly due to the similar densities of pure ethaline and pure propylene carbonate. As shown in Figure 1, no experimental data are available for the ethaline-propylene carbonate mixtures for *x*_DES_ > 0.2. On the basis of the excellent agreement between the MD and experiments for *x*_DES_ < 0.2 and for the rest of the ethaline-containing solvents, our new predictions can be considered trustworthy.

#### 3.1.2 Excess Chemical Potentials and Henry Coefficients

**Figure 2:**
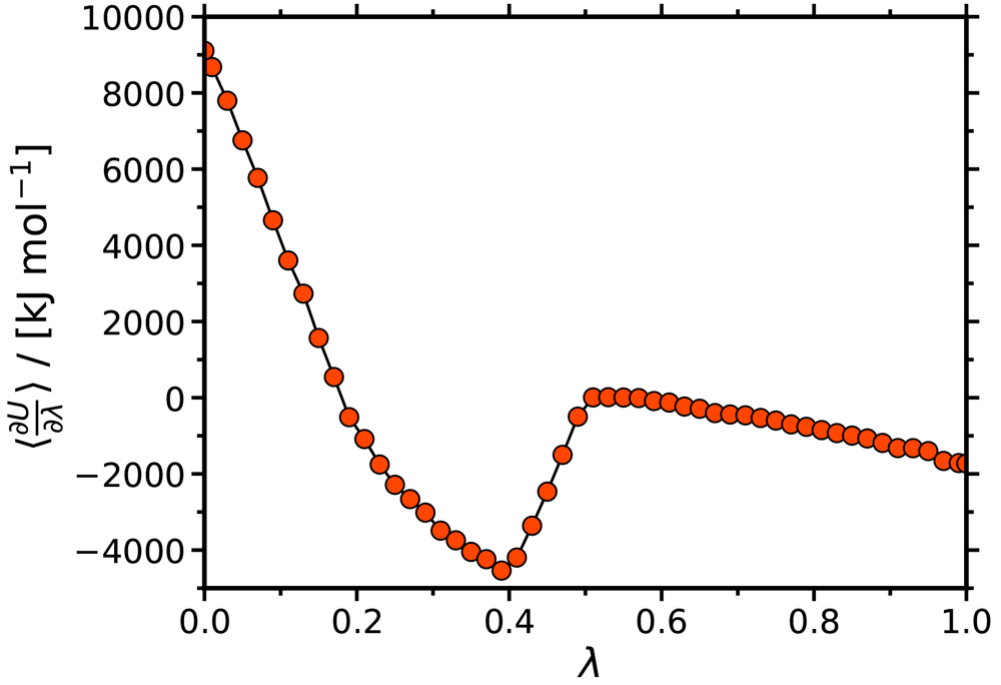
Average values of the partial derivative of the total energy with respect to the parameter λ as a function of λ for CO_2_ in methanol at *T* = 313.15 K and *P* = 2 atm. By construction, =0 at λ = 0.5.^63,67^ The line connecting the symbols is to guide the eye.

In this section, we present the computed excess chemical potentials and Henry coefficients of CO_2_, oxalic acid, and formic acid in different solvents consisting of a DES (i.e., reline or ethaline) and an organic cosolvent (methanol or propylene carbonate). Our approach was verified by comparing the solubility computed from MC simulations with experimental measurements for the case of CO_2_ in pure methanol at *T* = 313.15 K and 2 atm. Figure 2 shows the values of the average derivative of the energy with respect to the λ parameter in the CFCMC simulations as a function of λ. Using thermodynamic integration (eq 6), we obtain μ_CO_2__^ex^ = −3.27 kJ/mol, and from this we compute *H*_CO_2__ = 0.58 MPa. This value deviates by around 4% from the respective experimental Henry coefficient reported by Xia et al.^85^ This small deviation indicates that the chosen force fields and the method are reliable.

The computed values for μ^ex^ and Henry coefficient of CO_2_, oxalic acid, and formic acid in the different solvents are listed in Table 2. As can be seen, the solubility of CO_2_ in pure methanol and pure propylene carbonate is almost equal (absolute deviation of ca. 5%). Clearly, the addition of DES in these organic solvents reduces the CO_2_ solubilities. For *x*_DES_ = 0.4, the solubilities of CO_2_ are reduced by ca. 50% and 30% for reline-methanol and ethaline-methanol mixed solvents, respectively. For the same *x*_DES_ in ethaline-propylene carbonate mixture, the solubility of CO_2_ is reduced by ca. 20%. The Henry coefficients listed in Table 2 indicate that solvents containing ethaline are slightly better adsorbents of CO_2_ than reline-containing mixtures.

**Table 2:** Computed Excess Chemical Potentials μ^ex^ (Relative to an Ideal Gas, In Units of kJ/mol) and Henry Coefficients *H* (in units of MPa) of CO_2_, Oxalic Acid (OA), and Formic Acid (FA) in Different Solvents^a^

solvent	*x*_DES_	μ_CO_2__^ex^	*H*_CO_2__	μ_OA_^ex^	*H*_OA_	μ_FA_^ex^	*H*_FA_
reline-MeOH	0	–3.2	0.5	–50.5	2.7 × 10^–9^	–47.1	1.1 × 10^–8^
reline-MeOH	0.1	–3.0	0.6	–63.0	1.9 × 10^–11^	–43.1	6.1 × 10^–8^
reline-MeOH	0.2	–2.2	0.9	–61.3	4.1 × 10^–11^	–43.9	4.6 × 10^–8^
reline-MeOH	0.4	–1.6	1.3	–68.0	3.1 × 10^–12^	–42.2	1.0 × 10^–7^
reline-MeOH	1	2.4	7.9	–62.1	1.9 × 10^–9^	–45.7	5.2 × 10^–7^
ethaline-MeOH	0.1	–3.6	0.5	–62.6	2.2 × 10^–11^	–43.0	6.0 × 10^–8^
ethaline-MeOH	0.2	–2.8	0.7	–62.0	3.0 × 10^–11^	–42.8	6.9 × 10^–8^
ethaline-MeOH	0.4	–2.3	1.0	–66.0	6.5 × 10^–12^	–49.8	4.5 × 10^–9^
ethaline-MeOH	1	1.4	5.0	–64.1	8.3 × 10^–10^	–44.3	7.5 × 10^–7^
ethaline-PC	0	–3.5	0.7	–62.1	3.9 × 10^–11^	–38.8	4.7 × 10^–7^
ethaline-PC	0.1	–3.2	0.8	–66.5	6.5 × 10^–12^	–42.2	1.2 × 10^–7^
ethaline-PC	0.2	–3.5	0.7	–65.7	9.0 × 10^–12^	–42.4	1.1 × 10^–7^
ethaline-PC	0.4	–2.5	1.1	–70.0	1.6 × 10^–12^	–47.6	1.3 × 10^–8^
ethaline-PC	1	1.4	5.0	–64.1	8.3 × 10^–10^	–44.3	7.5 × 10^–7^

a The temperature *T* is 298.15 K for all solvents except for pure reline and pure ethaline for which *T* = 350.15 K. Pressure is equal to 1 atm for all systems.

Interestingly, the Henry coefficient of oxalic acid in DES-methanol mixtures is much lower compared to the one in the pure solvents (i.e., *x*_DES_ = 0 and 1). We speculate that this could be due to an interplay between hydrogen bonding interactions and a commensurate fit of the oxalic acid molecule in the liquid structure. Overall, the computed Henry coefficients show that adding a choline-based DES to the organic solvent increases the solubilities of oxalic acid and formic acid. While the solubility of CO_2_ is reduced as a result of adding a DES to methanol or propylene carbonate, it is important to note that the reduction is not very large and the Henry coefficients are still relatively high. The mixed solvents investigated here have higher CO_2_ Henry coefficients compared to aqueous solutions at the same conditions, which are typically used in electrochemical processes.^5^ This is an important finding since the design of an electrolyte with high CO_2_ solubility could potentially improve conversion rates by increasing the concentration of CO_2_ at the surface of the electrode.^15^

### 3.2 Transport Properties

#### 3.2.1 Viscosities

**Figure 3:**
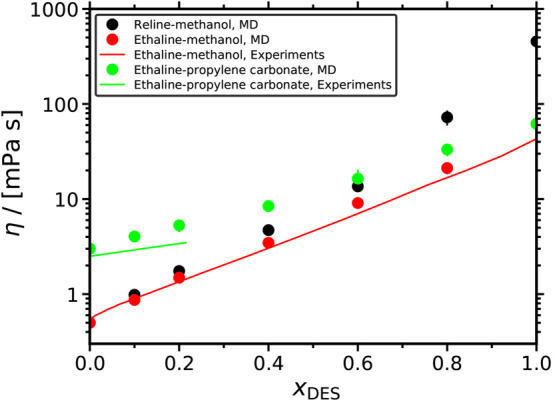
Viscosities of the ethaline-propylene carbonate, ethaline-methanol, and reline-methanol mixtures as a function of the mole fraction of DES at 298 K and 1 atm. The red and green lines represent experimental measurements by Wang et al.^86^ (ethaline-methanol) and by Zafarani-Moattar et al.^87^ (ethaline-propylene carbonate), respectively. The experimentally measured viscosity of pure reline is equal to 750 mPa s.^88^ Tabulated values of the computed viscosities along with their standard deviations are presented in the Supporting Information.

The computed viscosities of the ethaline-propylene carbonate, ethaline-methanol, and reline-methanol mixtures are shown in Figure 3 as a function of the DES content. Available experimental data^86−88^ are also shown in this figure along with the available experiments. Clearly, the viscosity increases with the DES content. Interestingly, for the ethaline-propylene carbonate mixture, this is the opposite behavior compared to the densities discussed earlier. The reason is that the density of pure propylene carbonate is slightly higher compared to pure ethaline, while the viscosity of propylene carbonate is much smaller than that of ethaline. This can be mainly attributed to the fact that, unlike pure propylene carbonate, pure ethaline has a strong hydrogen bonding network (this is discussed in detail in the following section). The viscosities of the pure organic solvents are predicted with deviations from experiments of ca. 7% and 20% for methanol and propylene carbonate, respectively. For all mixed solvents, the deviation between the computed and the experimentally measured viscosities increases with the addition of DES in the mixtures (absolute standard deviations range from ca. 7% to 44% and ca. 20% to 50% for ethaline-methanol and ethaline-propylene carbonate, respectively). Although these deviations seem rather high, the available experimental data are limited (e.g., no experimental data exist for the reline-methanol mixture) and the uncertainties in the computed values are quite large, ranging from 7 to 25% (due to the difficulty in sampling the slow dynamics caused by the relatively low temperature). It is also important to note that large deviations are reported between different experimental measurements of viscosities of DES. For example, different sources report viscosity values of pure reline in the range of 630–840 mPa s. For more details the reader is referred to the review paper by Smith et al.^25^ In absolute values, the predicted viscosities from MD simulations are satisfactory, while the qualitative behavior of the systems is captured accurately. Given the scarcity of experimentally measured viscosities for most of the mixtures considered here, our MD data can serve as a first set of predictions to aid the design of industrial processes and further motivate experimental efforts. To improve the accuracy of the computations, further modifications to the force fields, combining rules, and/or charge scaling should be considered. Such an investigation is beyond the scope of the present study.

As can be seen in Figure 3, reline-methanol mixtures are significantly more viscous than ethaline-methanol for the whole DES composition range. For *x*_DES_ = 0.8, the viscosity of reline-methanol is higher than the viscosity of ethaline-methanol by almost a factor of 3. This is not surprising since pure reline is significantly more viscous than ethaline (i.e., η_reline_ = 455 mPa s and η_ethaline_ = 62 mPa s at 298 K). For *x*_DES_ < 0.2, the viscosities of both mixtures are within the same order of magnitude. In the range (*x*_DES_ ≤ 0.6), ethaline-propylene carbonate mixtures exhibit the largest viscosity. For *x*_DES_ > 0.8, reline-methanol viscosities are the highest, and ethaline-propylene carbonate viscosities become comparable to the viscosities of ethaline-methanol.

Overall, our results reveal that the addition of DES to the organic solvents have a very strong effect on the viscosities. From a practical point of view for electrochemical processes, this finding dictates the careful selection of the composition of the mixtures since large viscosities can limit mass transport and, thus, reduce the current density of the electrolytes.^31^ To this end, DES with relatively low viscosities such as reline or ethaline (or other) can be promising.

#### 3.2.2 Self-Diffusivities

As slow diffusion rates can be a limiting factor in electrochemical processes, it is essential to integrate an electrolyte that yields sufficient mass transfer of the reactants and products to and from the catalyst.^4,15,89^ Since no experimental diffusivity data are available for the mixtures studied here, our results are the first step toward the screening of solvents for an optimum electrolyte containing DES, methanol, and propylene carbonate. In this section, we present the computed self-diffusivities of all the species in the DES–organic solvent mixtures, and the self-diffusivities of infinitely diluted solutes (CO_2_, oxalic acid, and formic acid) in these mixtures.

**Figure 4:**
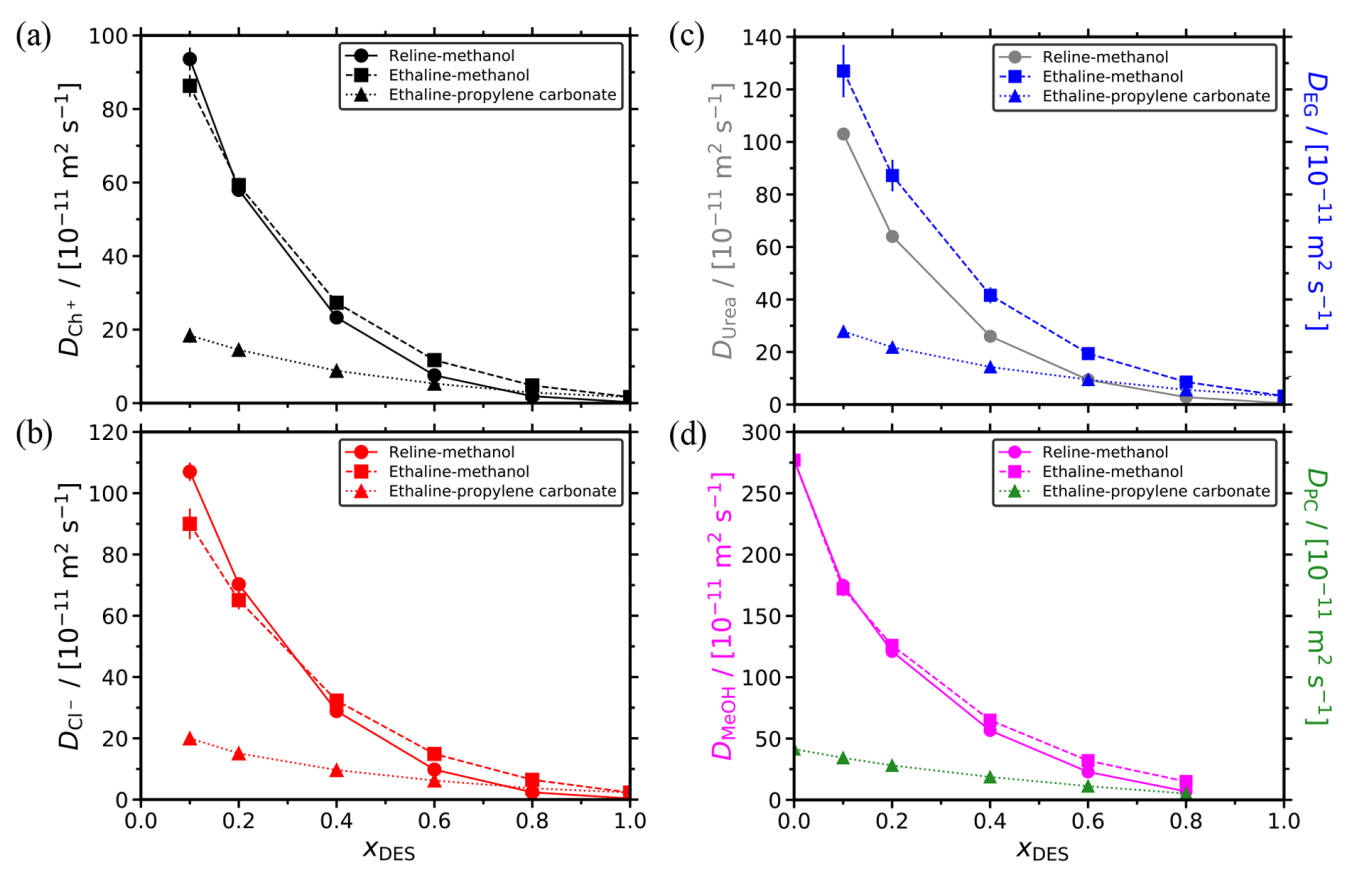
Self-diffusivities of all molecular species in the reline-methanol, ethaline-methanol, and ethaline-propylene carbonate mixtures as a function of the mole fraction of the DES at 298 K and 1 atm. (a) Choline (Ch^+^), (b) chloride (Cl^–^), and (c) HBDs. Urea and ethylene glycol (EG), and (d) organic solvents: methanol (MeOH) and propylene carbonate (PC). All computed diffusivities were corrected for finite-size effects using eq 10. The lines connecting the symbols are shown to guide the eye. Tabulated values along with their standard deviations are presented in the Supporting Information.

The computed self-diffusion coefficients of the different molecular species in the reline-methanol, ethaline-methanol, and ethaline-propylene carbonate mixed solvents are shown in Figure 4. All reported diffusivities were corrected for finite-size effects using eq 10. As can be clearly seen, the self-diffusivities of all components monotonically decrease as the DES composition increases. This is mainly due to the increasing viscosities of the mixtures upon the addition of DES (see Figure 3), resulting in reduced mobilities of the different species. Due to the very high viscosity of propylene carbonate, the ethaline-propylene carbonate mixtures are the most viscous for *x*_DES_ < 0.8. This is clearly reflected to the self-diffusivities of all species in this mixture for the same range of DES compositions, which have lower values compared to the methanol-containing solvents. The difference of all diffusivities in the ethaline-propylene carbonate and methanol-based mixtures becomes very pronounced at low DES concentrations. For example, at *x*_DES_ = 0.1, the diffusivity of ethylene glycol (i.e, the HBD) is ca. 4.5 times faster in ethaline-methanol than in ethaline-propylene carbonate. For high DES contents (i.e., *x*_DES_ ≥ 0.6), the differences between the self-diffusivities of the individual components in the different solvents becomes rather low. For such *x*_DES_, the diffusivities of both HBD, HBA, and organic components are very similar for the mixtures of reline-methanol and ethaline-propylene carbonate. The respective diffusivities in the ethaline-methanol solvent are the highest (but of similar magnitude), following the (opposite) viscosity trend.

**Figure 5:**
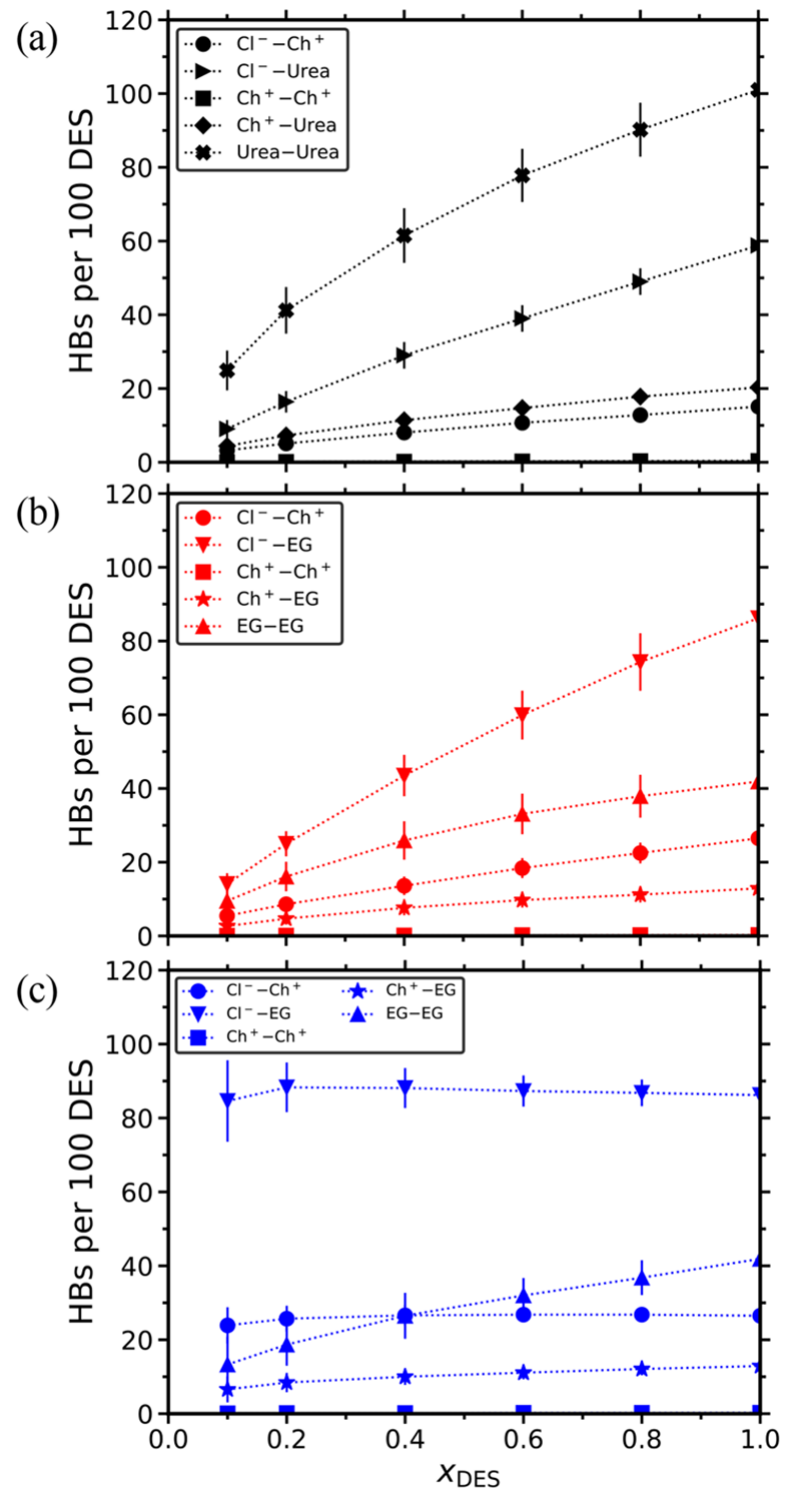
Number of hydrogen bonds (HBs) between HBD and HBA as a function of the mole fraction of DES for the (a) reline-methanol, (b) ethaline-methanol, and (c) ethaline-propylene carbonate mixtures at 298 K and 1 atm. The number of HBs is normalized to represent a system containing 100 DES molecules (i.e., 50 Ch^+^, 50 Cl^–^, and 100 urea or EG molecules). The dotted lines connecting the symbols are to guide the eye. Tabulated values along with their standard deviations are presented in the Supporting Information.

**Figure 6:**
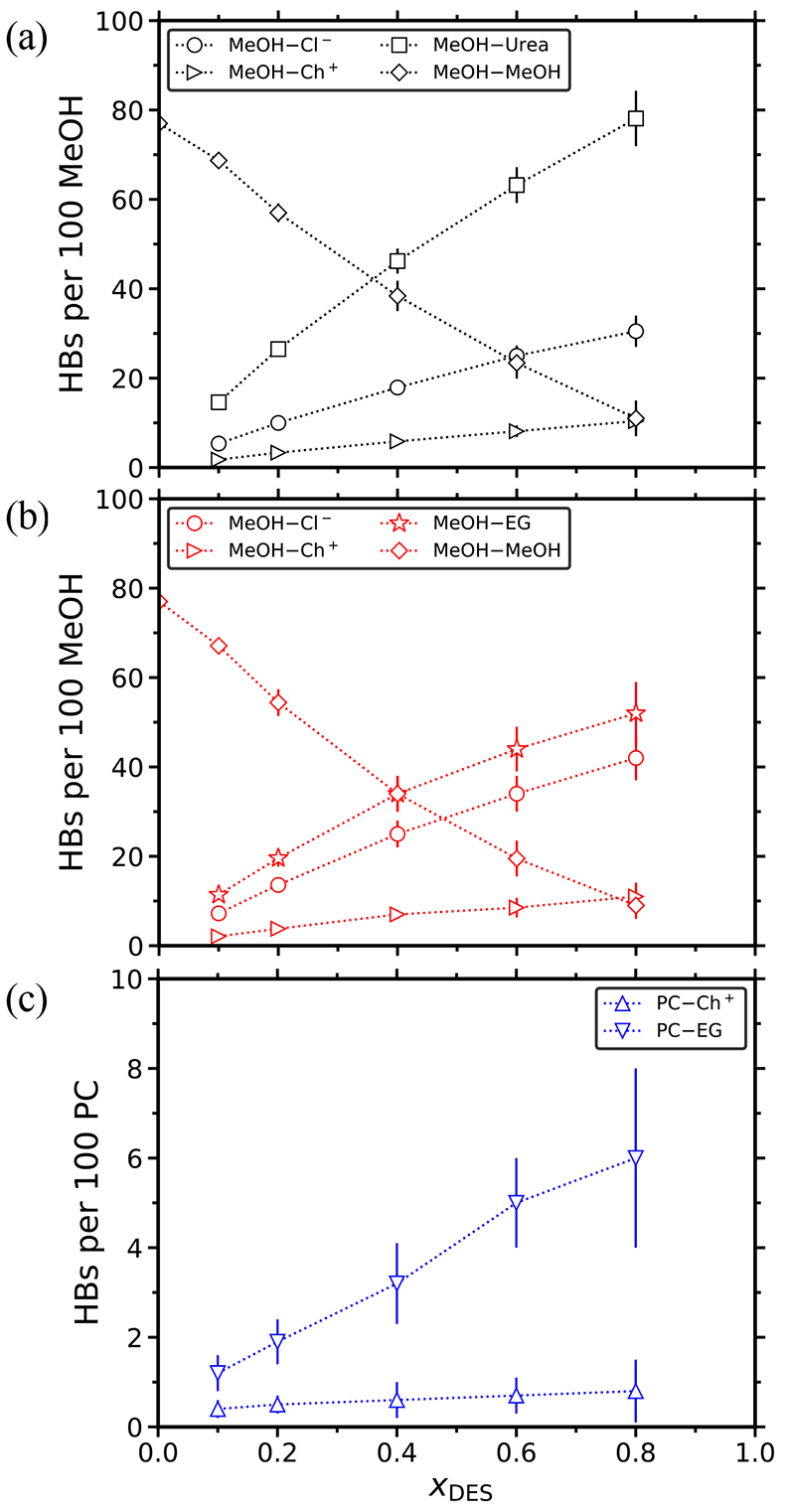
Number of hydrogen bonds (HBs) between HBD, HBA, and the organic components (i.e., MeOH or PC) as a function of the mole fraction of DES for the (a) reline-methanol, (b) ethaline-methanol, and (c) ethaline-propylene carbonate mixtures at 298 K and 1 atm. The number of HBs is normalized to represent a system containing 100 molecules of MeOH or PC. The dotted lines connecting the symbols are to guide the eye. Tabulated values along with their standard deviations are presented in the Supporting Information.

The molecular weight (MW) and the hydrodynamic radius are known factors to greatly affect the diffusivity of a molecule in a solvent.^90^ In DES (and DES-containing mixtures), the presence of an extended network of HBs is another crucial factor affecting mass transport.^26,56,57^ Choline, which is the heaviest species (MW ≈ 104.2 g/mol) among all HBDs and HBAs, has the lowest diffusion coefficient in all mixtures and DES compositions. Interestingly, the diffusivity of the much lighter chloride (MW ≈ 35.5 g/mol) is comparable to that of choline and lower than the diffusivities of both the HBD (i.e., urea and ethylene glycol with MW of 60.06 and 62.07 g/mol, respectively). In ethaline-containing mixtures, the diffusivity of ethylene glycol is higher than that of chloride by ca. 28%. In reline-methanol, the diffusivity of urea is slightly higher than that of chloride. Similar trends for the diffusivities of the HBD and HBA species were observed in the study by Celebi et al.^26^ for aqueous DES mixtures. This behavior can be explained by the HB network within the DES. As suggested by Perkins et al.,^56^ the fact that urea diffuses faster than most of the components in reline (despite having almost twice the MW of chloride) can be attributed to the formation of many HBs with other urea molecules and the anions. This can be clearly seen in Figure 5a, in which the computed HBs between the components of the DES are shown. Due to the varying number of molecules used in the MD simulations of different solvents (see Table 1), the number of HBs were normalized to represent a system containing 100 DES molecules. The number of the organic molecules follows from *x*_DES_. As shown in Figure 5, in the methanol-containing solvents all HB combinations monotonically increase as more DES is added to the mixture. The number of HBs formed between the various species increases ca. 2 to 6 times in the range of *x*_DES_ = 0.1–1. In the reline-methanol mixture, the rise in the number of urea–urea HBs is impressive, going from 25 to 101 (per 100 reline molecules). In the ethaline-methanol mixture, the anion-HBD HBs are also significantly increased, ranging from 14 to 86 (per 100 ethaline molecules) in the range of *x*_DES_ = 0.1–1. In the same mixture, the HBs between the HBD molcules are more than quadrupled (10 to 42/100 DES). The gradual development of this strong HB network is the main reason for the increasing viscosities and decreasing diffusivities of the different species in the methanol-containing solvents discussed earlier. In ethaline-propylene carbonate, the numbers of HBs formed between the various species do not significantly vary with *x*_DES_. This is mainly due to the lack of HB formation between the organic component and most of the DES species. The computed number of HBs formed between the organic solvents and the DES species are shown in Figure 6. Again, the number of HBs is normalized to represent a system containing 100 methanol or propylene carbonate molecules. The number of HBD and HBA follows from *x*_DES_. In contrast to propylene carbonate, methanol can form HBs with all the DES components (and with other methanol molecules). Thus, as *x*_DES_ increases, the methanol-methanol HBs are being depleted, and methanol forms HBs with the HBDs and HBAs. As can be clearly seen in Figure 6a,b, methanol primarily forms HBs with the HBD (urea or ethylene glycol) and secondarily with the anions. This HB behavior, combined with the relatively low MW of methanol (≈32 g/mol), are the main reasons for the fast self-diffusivities shown in Figure 4d. The lack of HBs between propylene carbonate and most of the DES components can be seen directly in Figure 6c and indirectly in Figure 5c. In the latter, the absence of competition between the organic component and the DES species to form HBs is the main reason for the almost constant HBs numbers between the HBA and HBD of the ethaline, with the only exception being the increasing HBD–HBD HBs.

**Figure 7:**
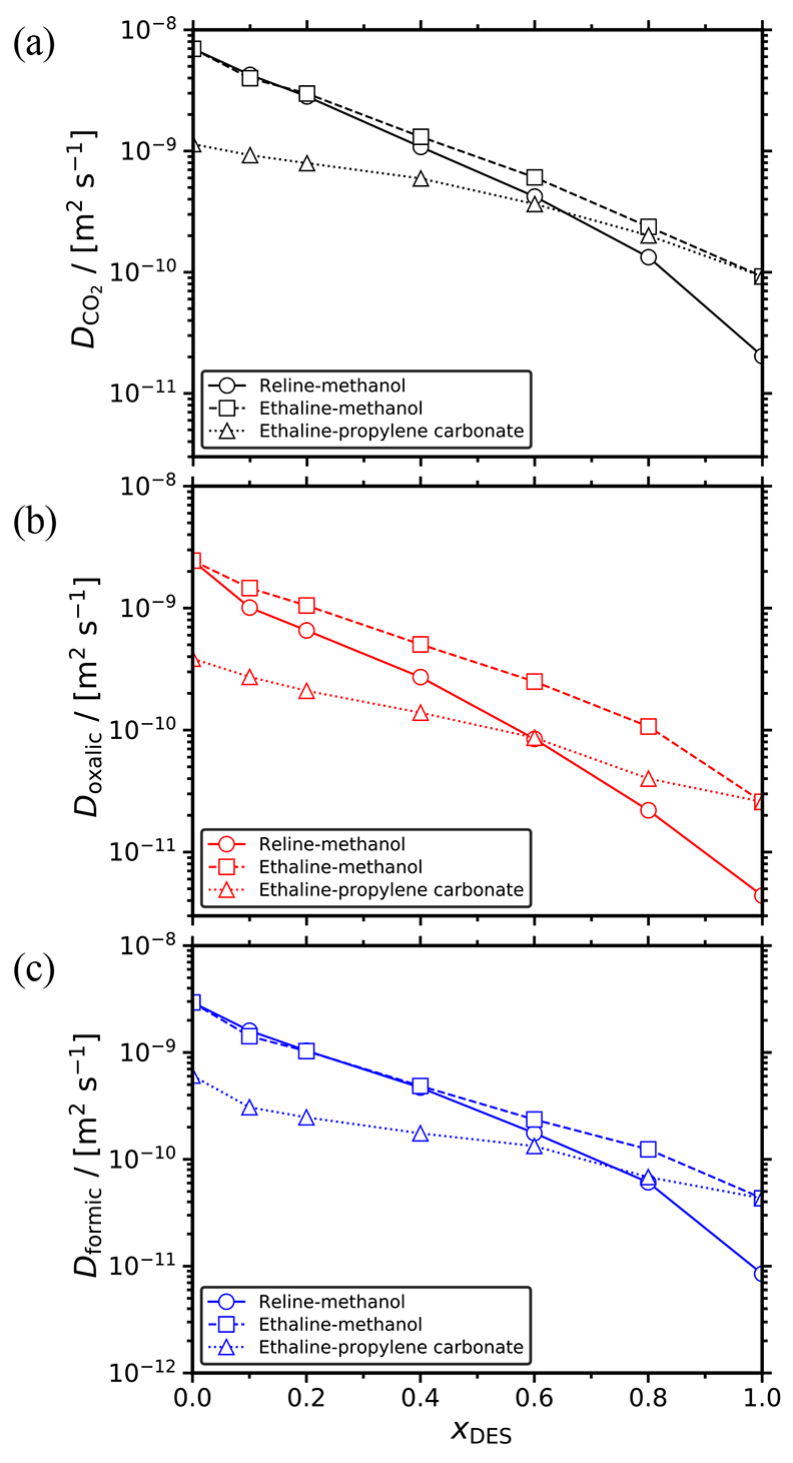
Self-diffusivities of infinitely diluted (a) CO_2_, (b) oxalic acid, and (c) formic acid in the reline-methanol, ethaline-methanol, and ethaline-propylene carbonate mixtures as a function of the mole fraction of DES at 298 K and 1 atm. All computed diffusivities were corrected for finite-size effects using eq 10. The error bars are smaller than the symbols size. The lines connecting the symbols are shown to guide the eye. Tabulated values along with their standard deviations are presented in the Supporting Information.

The self-diffusion coefficients of infinitely diluted CO_2_, oxalic acid, and formic acid in the different solvents are shown in Figure 7 as a function of *x*_DES_. Consistently with our findings for the solvents, the diffusivities of all solutes decrease as the DES mole fraction increases. In all mixtures, CO_2_ has the highest self-diffusivity followed by formic acid and oxalic acid. This order is in line with the molecular weights of these solutes. The highest diffusivities of all solutes are observed in ethaline-methanol. For *x*_DES_ < 0.4, all solutes diffuse faster in the methanol-based solvents than in the ethaline-propylene carbonate mixture. As discussed earlier, this can be mainly attributed to the high viscosity of the ethaline-propylene carbonate mixture for this composition range. At *x*_DES_ = 0.6, the lines representing the self-diffusivities of CO_2_ (Figure 7a), oxalic (Figure 7b), and formic acid (Figure 7c) in reline-methanol intersect with the respective lines showing the diffusivities in ethaline-propylene carbonate. For *x*_DES_ > 0.6, the diffusivities of the solutes in reline-methanol become the slowest due to the fact that this mixture is the most viscous one in this concentration range as shown in Figure 3. Because of the very small number of solutes used in the MD simulations (corresponding to infinite dilution), a solute-DES or solute-organic solvent HB analysis is not a very accurate descriptor for explaining the diffusivity behavior of the solutes, thus these HBs are not reported here.

#### 3.2.3 Ionic Conductivities

Another important property to optimize when designing electrolytes for electrochemical applications is ionic conductivity since electrolytes ensuring fast electron transfer are essential for high-performance electrochemical conversions. Recently, ionic liquid-based electrolyte solutions have been studied for the electroreduction of CO_2_ to valued-added products.^5^ To the best of our knowledge, no experimental data are available for the ionic conductivities of the DES-organic solvent mixtures considered here. The ionic conductivity of pure reline has been measured experimentally by various groups to be in the range of 0.024–0.764 S m^–1^ for *T* = 293–353 K, respectively.^45,46,91,92^ It is important to note that the actual values reported in literature significantly vary depending on the experimental technique used and the purity of the DES. For example, Agieienko and Buchner^45^ reported an electric conductivity of 0.024 S m^–1^ for pure reline at 298 K, while at the same conditions, Mjalli and Ahmed^46^ report a value of 0.18 S m^–1^, which is an order of magnitude higher. Celebi et al.^26^ reported a value of 0.09 S m^–1^ computed in MD simulations at 303 K. Here, a value of 0.11 S m^–1^ has been computed for *T* = 298 K. The measured ionic conductivity of ethaline ranges from ca. 0.62 to 2.08 S m^–1^ in the temperature range of 293–353 K.^46,92^ At room temperature it is equal to ca. 0.70 S m^–1^ (the exact value depends on the experimental study). Here, we computed a value of 0.63 S m^–1^, which is in reasonable agreement with the experiments. Since a thorough validation of the computed conductivities for the mixtures of DES with methanol and propylene carbonate is not possible due to the absence of experimental measurements and due to the fact that the NE equation has been shown to slightly overpredict conductivities,^54,80,93^ our results should be interpreted mostly qualitatively.

**Figure 8:**
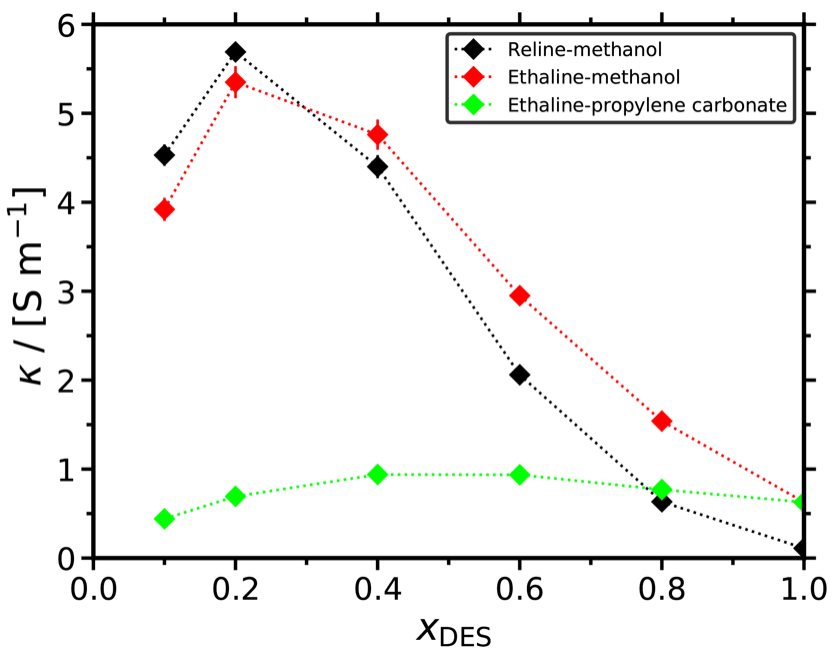
Ionic conductivities of the reline-methanol, ethaline-methanol, and ethaline-propylene carbonate mixtures as a function of the mole fraction of DES at 298 K and 1 atm. The dotted lines connecting the symbols are to guide the eye. Tabulated values of the computed ionic conductivities along with their standard deviations are presented in the Supporting Information.

The computed ionic conductivities of all mixed solvents studied in this work are shown in Figure 8 as a function of *x*_DES_. For all solvents, the ionic conductivities exhibit a nonmonotonic behavior. As *x*_DES_ increases, the ionic conductivities initially increase until a maximum value after which a sharp decline is observed. This can be explained by the fact that as ionic content (i.e., DES) is added to the mixture, the ionic conductivity initially increases up to the maximum value. However, the sharp increase of the viscosity due to the formation of the strong HB network within the DES (see Figures 5 and 6) restricts the mobility of the ions, causing the decline of κ after a certain *x*_DES_. This nonmonotonic behavior is fully consistent with the MD data by Celebi et al.^26^ and the experiments by Agieienko et al.^45^ for aqueous reline and ethaline solutions. Mjalli and Ahmed^46^ also observed this behavior for reline-ethaline mixtures.

Methanol-containing solvents have higher ionic conductivities compared to ethaline-propylene carbonate. For *x*_DES_ ≤ 0.6, this difference is significant, i.e., a factor of 2 to 6. The only exception is for *x*_DES_ = 0.8, for which the ethaline-propylene carbonate solvent exhibits slightly higher ionic conductivity than the reline-methanol one. This is in-line with the viscosity of these mixtures, which follows the exact same trend. The maximum electric conductivities are at *x*_DES_ = 0.2 for both the methanol-containing mixtures and at 0.4 ≤ *x*_DES_ ≤ 0.6 for ethaline–propylene carbonate. Since the increase in mobility by diluting ethaline with propylene carbonate is much lower compared to methanol (i.e., the slopes of the diffusivity curves in Figures 4 and 7), the peak of ionic conductivity for ethaline-propylene carbonate is shifted toward higher *x*_DES_. As *x*_DES_ approaches 1, the hydrogen-bonding network in the DES becomes extensive, causing the viscosity to significantly increase and, thus, the ionic conductivities of all solvents to reach their minimum. The only exception is ethaline-propylene carbonate, due to the very high viscosity of the pure organic component.

## 4 Conclusions

The electrochemical reduction of CO_2_ to value-added products, such as formic and oxalic acid, is considered to be a promising carbon utilization route for partially mitigating the greenhouse effect. Recently, DES have been considered as possible electrolytes for the reduction reactions of CO_2_ as a nontoxic and cost-efficient alternative to ionic liquids. Despite the distinct advantages of these solvents, the applicability of pure DES as electrolytes is hindered by high viscosities. Mixtures of DES with organic solvents can be a promising way of designing superior electrolytes by exploiting the advantages of each solvent type. In this study, the Henry coefficients and self-diffusivities of CO_2_, oxalic acid, and formic acid in reline-methanol, ethaline-methanol, and ethaline-propylene carbonate mixed solvents were computed using MC and MD simulations. The densities, viscosities, self-diffusivities, and ionic conductivities of the mixed solvents were also computed. The simulations were performed at *T* = 298 K, *P* = 1 atm, and mixture compositions *x*_DES_ = [0,1]. Our simulations showed that the Henry coefficients of CO_2_ in the ethaline-methanol and ethaline-propylene carbonate mixtures are in the same order of magnitude as the pure organic components. The reline-methanol mixtures were found to have slightly lower affinity toward CO_2_. Overall, the addition of DES to the organic solvents was found to increase the solubilities of oxalic and formic acids. The densities and viscosities of the mixed solvents monotonically increase with the mole fraction of DES. The only exception was observed for the density of ethaline-propylene carbonate which shows the opposite behavior due to the fact that the pure organic component is much denser than the pure DES. The self-diffusivities of all components (i.e., HBDs, HBAs, methanol, and propylene carbonate) in the mixtures significantly decrease as the mole fraction of DES increases. Interestingly, the self-diffusivities of the infinitely diluted CO_2_ and oxalic and formic acids decrease by 1 to 2 orders of magnitude as the composition of the mixture shifts from the pure organic component to pure DES. Our HB analysis revealed that the number of HBs between the DES species is vastly affected by the presence of methanol. As the mole fraction of DES increases, the HBs formed between methanol molecules are being depleted and methanol starts forming new HBs with the HBAs and HBDs of reline. At the same time, a sharp increase in the HBD-HBD and HBD-anion is observed. In sharp contrast, the presence of propylene carbonate has a smaller effect on the HB network of the DES, since it cannot form HBs with most of the DES species. A nonmonotonic behavior was observed for the computed ionic conductivities as a function of composition, which initially increased with the mole fraction of DES, showed a peak at a specific mole fraction for each mixture, and then decreased as more DES was added to the mixture. This finding is in-line with prior literature studies on aqueous DES solutions and mixtures of reline with ethaline. From an application point of view, the thermophysical data produced in this study suggests that the mixtures with low DES content could be the most practical in electrochemical processes since these mixtures exhibit lower viscosities compared to pure DES, higher ionic conductivities than the pure organic solvents, and good absorption capabilities. For most of the mixtures studied here, no prior experimental measurements exist, thus our findings can be considered a first approach based on which further experimental and theoretical studies of DES containing electrolyte solutions can be performed.

## Supporting Information Available

Force field parameters and tabulated raw data for the computed thermo-physical properties (PDF)

## References

[ref1] Mac Dowell N.; Fennell P. S.; Shah N.; Maitland G. C. The role of CO_2_ capture and utilization in mitigating climate change. Nature Climate Change 2017, 7, 243–249. 10.1038/nclimate3231.

[ref2] Centi G.; Perathoner S. Opportunities and prospects in the chemical recycling of carbon dioxide to fuels. Catal. Today 2009, 148, 191–205. 10.1016/j.cattod.2009.07.075.

[ref3] Whipple D. T.; Kenis P. J. Prospects of CO_2_ utilization via direct heterogeneous electrochemical reduction. J. Phys. Chem. Lett. 2010, 1, 3451–3458. 10.1021/jz1012627.

[ref4] Vasileff A.; Zheng Y.; Qiao S. Z. Carbon solving carbonas problems: recent progress of nanostructured carbon-based catalysts for the electrochemical reduction of CO_2_. Adv. Energy Materials 2017, 7, 1700759 10.1002/aenm.201700759.

[ref5] Yang D.; Zhu Q.; Han B. Electroreduction of CO_2_ in ionic liquid-based electrolytes. Innovation 2020, 1, 100016 10.1016/j.xinn.2020.100016. 34557704 PMC8454664

[ref6] Hu B.; Guild C.; Suib S. L. Thermal, electrochemical, and photochemical conversion of CO_2_ to fuels and value-added products. Journal of CO_2_ Utilization 2013, 1, 18–27. 10.1016/j.jcou.2013.03.004.

[ref7] Ramdin M.; De Mot B.; Morrison A. R. T.; Breugelmans T.; van den Broeke L. J. P.; Trusler J. P. M.; Kortlever R.; de Jong W.; Moultos O. A.; Xiao P.; Webley P. A.; Vlugt T. J. H. Electroreduction of CO_2_/CO to C_2_ Products: Process Modeling, Downstream Separation, System Integration, and Economic Analysis. Ind. Eng. Chem. Res. 2021, 60, 17862–17880. 10.1021/acs.iecr.1c03592. 34937989 PMC8679093

[ref8] Spinner N. S.; Vega J. A.; Mustain W. E. Recent progress in the electrochemical conversion and utilization of CO_2_. Catal. Sci. Technol. 2012, 2, 19–28. 10.1039/C1CY00314C.

[ref9] Hussin F.; Aroua M. K. Recent development in the electrochemical conversion of carbon dioxide: Short review. AIP Conf. Proc. 2018, 2124, 030017 10.1063/1.5117139.

[ref10] Ramdin M.; Morrison A. R. T.; de Groen M.; van Haperen R.; de Kler R.; Irtem E.; Laitinen A. T.; van den Broeke L. J. P.; Breugelmans T.; Trusler J. P. M.; Jong W. d.; Vlugt T. J. H. High-Pressure Electrochemical Reduction of CO_2_ to Formic Acid/Formate: Effect of pH on the Downstream Separation Process and Economics. Ind. Eng. Chem. Res. 2019, 58, 22718–22740. 10.1021/acs.iecr.9b03970. PMC6369647 30774193

[ref11] Global Formic Acid Market Report, History and Forecast. https://dataintelo.com/report/formic-acid-market/, Date accessed: 15 February 2022.

[ref12] Oxalic acid: compound summary. https://pubchem.ncbi.nlm.nih.gov/compound/Oxalic-acid, Date accessed: 15 February 2022.

[ref13] Oxalic acid specific uses. http://www.lubonchem.com/blog/?p=993, Date accessed: 15 February 2022.

[ref14] Zhang M. High Quality Best Price 99.6% Oxalic Acid from China Largest Manufacturer. https://www.alibaba.com/product-detail/Price-Oxalic-Acid-Oxalic-Acid-Price_60855082411.html?spm/a2700.7724857.topad_classic.d_title.737f6796Z67cZy, Date accessed: 15 February 2022.

[ref15] Jhong H.-R. M.; Ma S.; Kenis P. J. A. Electrochemical conversion of CO_2_ to useful chemicals: current status, remaining challenges, and future opportunities. Curr. Opin. Chem. Eng. 2013, 2, 191–199. 10.1016/j.coche.2013.03.005.

[ref16] Thorson M. R.; Siil K. I.; Kenis P. J. Effect of cations on the electrochemical conversion of CO_2_ to CO. J. Electrochem. Soc. 2013, 160, F69 10.1149/2.052301jes.

[ref17] Lim H.-K.; Kim H. The mechanism of room–temperature ionic-liquid-based electrochemical CO_2_ reduction: a review. Molecules 2017, 22, 536 10.3390/molecules22040536. PMC6154551 28350332

[ref18] Tomita Y.; Teruya S.; Koga O.; Hori Y. Electrochemical reduction of carbon dioxide at a platinum electrode in acetonitrile-water mixtures. J. Electrochem. Soc. 2000, 147, 4164 10.1149/1.1394035.

[ref19] Hansen B. B.; Spittle S.; Chen B.; Poe D.; Zhang Y.; Klein J. M.; Horton A.; Adhikari L.; Zelovich T.; Doherty B. W.; Gurkan B.; Maginn E. J.; Ragauskas A.; Dadmun M.; Zawodzinski T. A.; Baker G. A.; Tuckerman M. E.; Savinell R. F.; Sangoro J. R. Deep Eutectic Solvents: A Review of Fundamentals and Applications. Chem. Rev. 2021, 121, 1232 10.1021/acs.chemrev.0c00385. 33315380

[ref20] Sarmad S.; Xie Y.; Mikkola J. P.; Ji X. Screening of Deep Eutectic Solvents (DESs) as Green CO_2_ Sorbents: From Solubility to Viscosity. New J. Chem. 2017, 41, 290 10.1039/C6NJ03140D.

[ref21] Salehi H. S.; Hens R.; Moultos O. A.; Vlugt T. J. H. Computation of gas solubilities in choline chloride urea and choline chloride ethylene glycol Deep Eutectic Solvents using Monte Carlo simulations. J. Mol. Liq. 2020, 316, 113729 10.1016/j.molliq.2020.113729.

[ref22] Salehi H. S.; Hens R.; Moultos O. A.; Vlugt T. J. H. Computation of Gas Solubilities in Choline Chloride Urea and Choline Chloride Ethylene Glycol Deep Eutectic Solvents Using Monte Carlo Simulations. J. Mol. Liq. 2020, 316, 113729 10.1016/j.molliq.2020.113729.

[ref23] Velez C.; Acevedo O. Simulation of deep eutectic solvents: Progress to promises. WIREs Comput. Mol. Sci. [Online early access]. e1598 10.1002/wcms.1598. Published online: 2022-01-11 (accessed 2022-02-09).

[ref24] Salehi H. S.; Polat H. M.; de Meyer F.; Houriez C.; Coquelet C.; Vlugt T. J. H.; Moultos O. A. Vapor pressures and vapor phase compositions of choline chloride urea and choline chloride ethylene glycol deep eutectic solvents from molecular simulation. J. Chem. Phys. 2021, 155, 114504 10.1063/5.0062408. 34551525

[ref25] Smith E. L.; Abbott A. P.; Ryder K. S. Deep Eutectic Solvents (DESs) and their Applications. Chem. Rev. 2014, 114, 11060 10.1021/cr300162p. 25300631

[ref26] Celebi A. T.; Vlugt T. J. H.; Moultos O. A. Structural, Thermodynamic and Transport Properties of Aqueous Reline and Ethaline Solutions from Molecular Dynamics Simulations. J. Phys. Chem. B 2019, 123, 11014–11025. 10.1021/acs.jpcb.9b09729. 31794220 PMC6935864

[ref27] Celebi A. T.; Vlugt T. J. H.; Moultos O. A. Thermal Conductivity of Aqueous Solutions of Reline, Ethaline, and Glyceline Deep Eutectic Solvents; a Molecular Dynamics Simulation Study. Mol. Phys. 2021, 119, 1876263 10.1080/00268976.2021.1876263.

[ref28] Salehi H. S.; Moultos O. A.; Vlugt T. J. H. Interfacial Properties of Hydrophobic Deep Eutectic Solvents with Water. J. Phys. Chem. B 2021, 125, 12303–12314. 10.1021/acs.jpcb.1c07796. 34719232 PMC8591605

[ref29] Zhang L.; Siepmann J. Direct calculation of Henry’s law constants from Gibbs ensemble Monte Carlo simulations: nitrogen, oxygen, carbon dioxide and methane in ethanol. Theor. Chem. Acc. 2006, 115, 391–397. 10.1007/s00214-005-0073-1.

[ref30] Craveiro R.; Aroso I.; Flammia V.; Carvalho T.; Viciosa M.; Dionísio M.; Barreiros S.; Reis R.; Duarte A. R. C.; Paiva A. Properties and thermal behavior of natural deep eutectic solvents. J. Mol. Liq. 2016, 215, 534–540. 10.1016/j.molliq.2016.01.038.

[ref31] Vasilyev D. V.; Rudnev A. V.; Broekmann P.; Dyson P. J. A general and facile approach for the electrochemical reduction of carbon dioxide inspired by deep eutectic solvents. ChemSusChem 2019, 12, 1635–1639. 10.1002/cssc.201900579. 30811822

[ref32] Cheng D.; Negreiros F. R.; Aprà E.; Fortunelli A. Computational approaches to the chemical conversion of carbon dioxide. ChemSusChem 2013, 6, 944–965. 10.1002/cssc.201200872. 23716438

[ref33] Orozco G. A.; Moultos O. A.; Jiang H.; Economou I. G.; Panagiotopoulos A. Z. Molecular Simulation of Thermodynamic and Transport Properties for the H_2_O+NaCl System. J. Chem. Phys. 2014, 141, 234507 10.1063/1.4903928. 25527948

[ref34] Nezbeda I.; Moučka F.; Smith W. R. Recent progress in molecular simulation of aqueous electrolytes: Force fields, chemical potentials and solubility. Mol. Phys. 2016, 114, 1665–1690. 10.1080/00268976.2016.1165296.

[ref35] Jiang H.; Mester Z.; Moultos O. A.; Economou I. G.; Panagiotopoulos A. Z. Thermodynamic and Transport Properties of H_2_O + NaCl from Polarizable Force Fields. J. Chem. Theory Comput. 2015, 11, 3802–3810. 10.1021/acs.jctc.5b00421. 26574461

[ref36] Singh M. R.; Goodpaster J. D.; Weber A. Z.; Head-Gordon M.; Bell A. T. Mechanistic insights into electrochemical reduction of CO_2_ over Ag using density functional theory and transport models. Proc. Natl. Acad. Sci. U. S. A. 2017, 114, E8812–E8821. 10.1073/pnas.1713164114. 28973926 PMC5651780

[ref37] Döpke M. F.; Lützenkirchen J.; Moultos O. A.; Siboulet B.; Dufrêche J.-F.; Padding J. T.; Hartkamp R. Preferential Adsorption in Mixed Electrolytes Confined by Charged Amorphous Silica. J. Phys. Chem. C 2019, 123, 16711–16720. 10.1021/acs.jpcc.9b02975.

[ref38] Dwelle K. A.; Willard A. P. Constant Potential, Electrochemically Active Boundary Conditions for Electrochemical Simulation. J. Phys. Chem. C 2019, 123, 24095–24103. 10.1021/acs.jpcc.9b06635.

[ref39] Scalfi L.; Salanne M.; Rotenberg B. Molecular simulation of electrode-solution interfaces. Annu. Rev. Phys. Chem. 2021, 72, 189–212. 10.1146/annurev-physchem-090519-024042. 33395545

[ref40] Zhang Y.; Maginn E. J. Water-In-Salt LiTFSI Aqueous Electrolytes (2): Transport Properties and Li^+^ Dynamics Based on Molecular Dynamics Simulations. J. Phys. Chem. B 2021, 125, 13246–13254. 10.1021/acs.jpcb.1c07581. 34813336

[ref41] Kaneco S.; Iwao R.; Iiba K.; Ohta K.; Mizuno T. Electrochemical conversion of carbon dioxide to formic acid on Pb in KOH/methanol electrolyte at ambient temperature and pressure. Energy 1998, 23, 1107–1112. 10.1016/S0360-5442(98)00054-1.

[ref42] Kaneco S.; Iiba K.; Katsumata H.; Suzuki T.; Ohta K. Electrochemical reduction of high pressure CO_2_ at a Cu electrode in cold methanol. Electrochim. Acta 2006, 51, 4880–4885. 10.1016/j.electacta.2006.01.032.

[ref43] Fischer J.; Lehmann T.; Heitz E. The production of oxalic acid from CO_2_ and H_2_O. J. Appl. Electrochem. 1981, 11, 743–750. 10.1007/BF00615179.

[ref44] Ikeda S.; Takagi T.; Ito K. Selective formation of formic acid, oxalic acid, and carbon monoxide by electrochemical reduction of carbon dioxide. Bull. Chem. Soc. Jpn. 1987, 60, 2517–2522. 10.1246/bcsj.60.2517.

[ref45] Agieienko V.; Buchner R. Densities, Viscosities, and Electrical Conductivities of Pure Anhydrous Reline and Its Mixtures with Water in the Temperature Range (293.15 to 338.15) K. Journal of Chemical & Engineering Data 2019, 64, 4763–4774. 10.1021/acs.jced.9b00145.

[ref46] Mjalli F. S.; Ahmed O. U. Physical properties and intermolecular interaction of eutectic solvents binary mixtures: reline and ethaline. Asia-Pacific Journal of Chemical Engineering 2016, 11, 549–557. 10.1002/apj.1978.

[ref47] Potoff J. J.; Siepmann J. I. Vapor-liquid equilibria of mixtures containing alkanes, carbon dioxide, and nitrogen. AIChE J. 2001, 47, 1676–1682. 10.1002/aic.690470719.

[ref48] Chen B.; Potoff J. J.; Siepmann J. I. Monte Carlo calculations for alcohols and their mixtures with alkanes. Transferable potentials for phase equilibria. 5. United-atom description of primary, secondary, and tertiary alcohols. J. Phys. Chem. B 2001, 105, 3093–3104. 10.1021/jp003882x.

[ref49] Jorgensen W. L.; Maxwell D. S.; Tirado-Rives J. Development and testing of the OPLS all-atom force field on conformational energetics and properties of organic liquids. J. Am. Chem. Soc. 1996, 118, 11225–11236. 10.1021/ja9621760.

[ref50] Doherty B.; Acevedo O. OPLS Force Field for Choline Chloride-Based Deep Eutectic Solvents. J. Phys. Chem. B 2018, 122, 9982–9993. 10.1021/acs.jpcb.8b06647. 30125108

[ref51] Salas F. J.; Núñez-Rojas E.; Alejandre J. Stability of formic acid/pyridine and isonicotinamide/formamide cocrystals by molecular dynamics simulations. Theor. Chem. Acc. 2017, 136, 1–12. 10.1007/s00214-016-2024-4.

[ref52] Silva L. B.; Freitas L. C. G. Structural and thermodynamic properties of liquid ethylene carbonate and propylene carbonate by Monte Carlo Simulations. J. Mol. Struct. 2007, 806, 23–34. 10.1016/j.theochem.2006.10.014.

[ref53] Wang J.; Wolf R. M.; Caldwell J. W.; Kollman P. A.; Case D. A. Development and Testing of a General Amber Force Field. J. Comput. Chem. 2004, 25, 1157–1174. 10.1002/jcc.20035. 15116359

[ref54] Salehi H. S.; Celebi A. T.; Vlugt T. J. H.; Moultos O. A. Thermodynamic, transport, and structural properties of hydrophobic deep eutectic solvents composed of tetraalkylammonium chloride and decanoic acid. J. Chem. Phys. 2021, 154, 144502 10.1063/5.0047369. 33858163

[ref55] Perkins S. L.; Painter P.; Colina C. M. Experimental and Computational Studies of Choline Chloride-based Deep Eutectic Solvents. J. Chem. Eng. Data 2014, 59, 3652–3662. 10.1021/je500520h.

[ref56] Perkins S. L.; Painter P.; Colina C. M. Molecular Dynamic Simulations and Vibrational Analysis of an Ionic Liquid Analogue. J. Phys. Chem. B 2013, 117, 10250–10260. 10.1021/jp404619x. 23915257

[ref57] Celebi A. T.; Dawass N.; Moultos O. A.; Vlugt T. J. H. How sensitive are physical properties of choline chloride-urea mixtures to composition changes: Molecular dynamics simulations and Kirkwood-Buff theory. J. Chem. Phys. 2021, 154, 184502 10.1063/5.0049064. 34241035

[ref58] Frenkel D.; Smit B. Understanding molecular simulation: from algorithms to applications, 2nd ed.; Academic press: London, UK, 2002; Vol. 1.

[ref59] Prausnitz J. M.; Lichtenthaler R. N.; De Azevedo E. G. Molecular thermodynamics of fluid-phase equilibria, 3rd ed.; Pearson Education: Upper Saddle River, N.J, 1998.

[ref60] Hempel S.; Fischer J.; Paschek D.; Sadowski G. Activity Coefficients of Complex Molecules by Molecular Simulation and Gibbs-Duhem Integration. Soft Mater. 2012, 10, 26–41. 10.1080/1539445X.2011.599698.

[ref61] Rahbari A. Thermodynamics of Industrially Relevant Systems: Method Development and Applications. Ph.D. thesis, Delft University of Technology, Delft, The Netherlands, 2020.

[ref62] Shing K. S.; Gubbins K. E.; Lucas K. Henry constants in non-ideal fluid mixtures: computer simulation and theory. Mol. Phys. 1988, 65, 1235–1252. 10.1080/00268978800101731.

[ref63] Hens R.; Rahbari A.; Caro-Ortiz S.; Dawass N.; Erdös M.; Poursaeidesfahani A.; Salehi H.; Celebi A.; Ramdin M.; Moultos O. A.; Dubbeldam D.; Vlugt T. J. H. Brick–CFCMC: Open source software for Monte Carlo simulations of phase and reaction equilibria using the Continuous Fractional Component method. J. Chem. Inf. Model. 2020, 60, 2678–2682. 10.1021/acs.jcim.0c00334. 32275829 PMC7312392

[ref64] Polat H. M.; Salehi H. S.; Hens R.; Wasik D. O.; Rahbari A.; De Meyer F.; Houriez C.; Coquelet C.; Calero S.; Dubbeldam D.; Moultos O. A.; Vlugt T. J. H. New Features of the Open Source Monte Carlo Software Brick-CFCMC: Thermodynamic Integration and Hybrid Trial Moves. J. Chem. Inf. Model. 2021, 61, 3752–3757. 10.1021/acs.jcim.1c00652. 34383501 PMC8385706

[ref65] Shi W.; Maginn E. J. Continuous Fractional Component Monte Carlo: an adaptive biasing method for open system atomistic simulations. J. Chem. Theory Comput. 2007, 3, 1451–1463. 10.1021/ct7000039. 26633216

[ref66] Shi W.; Maginn E. J. Improvement in molecule exchange efficiency in Gibbs Ensemble Monte Carlo: development and implementation of the continuous fractional component move. J. Comput. Chem. 2008, 29, 2520–2530. 10.1002/jcc.20977. 18478586

[ref67] Rahbari A.; Hens R.; Ramdin M.; Moultos O. A.; Dubbeldam D.; Vlugt T. J. H. Recent advances in the Continuous Fractional Component Monte Carlo methodology. Mol. Simul. 2021, 47, 804–823. 10.1080/08927022.2020.1828585.

[ref68] Poursaeidesfahani A.; Torres-Knoop A.; Dubbeldam D.; Vlugt T. J. H. Direct free energy calculation in the Continuous Fractional Component Gibbs ensemble. J. Chem. Theory Comput. 2016, 12, 1481–1490. 10.1021/acs.jctc.5b01230. 26928892

[ref69] Rahbari A.; Hens R.; Nikolaidis I. K.; Poursaeidesfahani A.; Ramdin M.; Economou I. G.; Moultos O. A.; Dubbeldam D.; Vlugt T. J. H. Computation of partial molar properties using continuous fractional component Monte Carlo. Mol. Phys. 2018, 116, 3331–3344. 10.1080/00268976.2018.1451663.

[ref70] Plimpton S. Fast Parallel Algorithms for Short-Range Molecular Dynamics. J. Comput. Phys. 1995, 117, 1–19. 10.1006/jcph.1995.1039.

[ref71] Martínez L.; Andrade R.; Birgin E. G.; Martínez J. M. PACKMOL: A Package for Building Initial Configurations for Molecular Dynamics Simulations. J. Comput. Chem. 2009, 30, 2157–2164. 10.1002/jcc.21224. 19229944

[ref72] Jamali S. H.; Wolff L.; Becker T. M.; de Groen M.; Ramdin M.; Hartkamp R.; Bardow A.; Vlugt T. J. H.; Moultos O. A. OCTP: A Tool for On-the-fly Calculation of Transport Properties of Fluids with the Order-n Algorithm in LAMMPS. J. Chem. Inf. Model. 2019, 59, 1290–1294. 10.1021/acs.jcim.8b00939. 30742429

[ref73] Mondello M.; Grest G. S. Viscosity Calculations of n-Alkanes by Equilibrium Molecular Dynamics. J. Chem. Phys. 1997, 106, 9327 10.1063/1.474002.

[ref74] Yeh I.-C.; Hummer G. System-size Dependence of Diffusion Coefficients and Viscosities from Molecular Dynamics Simulations with Periodic Boundary Conditions. J. Phys. Chem. B 2004, 108, 15873–15879. 10.1021/jp0477147.

[ref75] Jamali S. H.; Bardow A.; Vlugt T. J. H.; Moultos O. A. Generalized Form for Finite-Size Corrections in Mutual Diffusion Coefficients of Multicomponent Mixtures Obtained from Equilibrium Molecular Dynamics Simulation. J. Chem. Theory Comput. 2020, 16, 3799–3806. 10.1021/acs.jctc.0c00268. 32338889 PMC7288667

[ref76] Celebi A. T.; Jamali S. H.; Bardow A.; Vlugt T. J. H.; Moultos O. A. Finite-size Effects of Diffusion Coefficients Computed from Molecular Dynamics: A Review of What we have Learned so far. Mol. Sim. 2021, 47, 831–845. 10.1080/08927022.2020.1810685.

[ref77] Jamali S. H.; Hartkamp R.; Bardas C.; Söhl J.; Vlugt T. J. H.; Moultos O. A. Shear viscosity computed from the finite-size effects of self-diffusivity in equilibrium molecular dynamics. J. Chem. Theory Comput. 2018, 14, 5959–5968. 10.1021/acs.jctc.8b00625. 30296092 PMC6236468

[ref78] Moultos O. A.; Zhang Y.; Tsimpanogiannis I. N.; Economou I. G.; Maginn E. J. System-size Corrections for Self-diffusion Coefficients Calculated from Molecular Dynamics Simulations: The Case of CO_2_, N-alkanes, and Poly (Ethylene Glycol) Dimethyl Ethers. J. Chem. Phys. 2016, 145, 074109 10.1063/1.4960776. 27544089

[ref79] Humbert M. T.; Zhang Y.; Maginn E. J. PyLAT: Python LAMMPS Analysis Tools. J. Chem. Inf. Model. 2019, 59, 1301–1305. 10.1021/acs.jcim.9b00066. 30844269

[ref80] Nordness O.; Brennecke J. F. Ion Dissociation in Ionic Liquids and Ionic Liquid Solutions. Chem. Rev. 2020, 120, 12873–12902. 10.1021/acs.chemrev.0c00373. 33026798

[ref81] Hayamizu K. Temperature dependence of self-diffusion coefficients of ions and solvents in ethylene carbonate, propylene carbonate, and diethyl carbonate single solutions and ethylene carbonate + diethyl carbonate binary solutions of LiPF 6 studied by NMR. J. Chem. Eng. Data 2012, 57, 2012–2017. 10.1021/je3003089.

[ref82] Humphrey W.; Dalke A.; Schulten K. VMD – Visual Molecular Dynamics. J. Mol. Graphics 1996, 14, 33–38. 10.1016/0263-7855(96)00018-5. 8744570

[ref83] Starr F. W.; Nielsen J. K.; Stanley H. E. Hydrogen-bond dynamics for the extended simple point-charge model of water. Phys. Rev. E 2000, 62, 579–587. 10.1103/PhysRevE.62.579. 11088494

[ref84] Erdös M.; Frangou M.; Vlugt T. J.; Moultos O. A. Diffusivity of α-, β-, γ-cyclodextrin and the inclusion complex of β-cyclodextrin: Ibuprofen in aqueous solutions; A molecular dynamics simulation study. Fluid Phase Equilib. 2021, 528, 112842 10.1016/j.fluid.2020.112842. 33024350 PMC7529625

[ref85] Xia J.; Jödecke M.; Pérez-Salado Kamps Á.; Maurer G. Solubility of CO_2_ in (CH_3_OH+ H_2_O). J. Chem. Eng. Data 2004, 49, 1756–1759. 10.1021/je049803i.

[ref86] Wang Y.; Ma C.; Liu C.; Lu X.; Feng X.; Ji X. Thermodynamic study of choline chloride-based deep eutectic solvents with water and methanol. J. Chem. Eng. Data 2020, 65, 2446–2457. 10.1021/acs.jced.9b01113.

[ref87] Zafarani-Moattar M. T.; Shekaari H.; Sadrmousavi Dizaj A. Investigation of solute–solvent interactions in binary and quaternary solutions containing lithium perchlorate, propylene carbonate, and the deep eutectic solvent (choline chloride/ethylene glycol) at T = (288.15 to 318.15) K. J. Mol. Liq. 2020, 319, 114090 10.1016/j.molliq.2020.114090.

[ref88] D’Agostino C.; Harris R. C.; Abbott A. P.; Gladden L. F.; Mantle M. D. Molecular motion and ion diffusion in choline chloride based deep eutectic solvents studied by 1 H pulsed field gradient NMR spectroscopy. Phys. Chem. Chem. Phys. 2011, 13, 21383–21391. 10.1039/c1cp22554e. 22033601

[ref89] Rudnev A. V.; Fu Y.-C.; Gjuroski I.; Stricker F.; Furrer J.; Kovács N.; Vesztergom S.; Broekmann P. Transport matters: boosting CO_2_ electroreduction in mixtures of [BMIm][BF_4_]/water by enhanced diffusion. ChemPhysChem 2017, 18, 3153–3162. 10.1002/cphc.201700737. 28872751

[ref90] Cussler E. L. Diffusion: Mass Transfer in Fluid Systems, 3rd ed.; Cambridge University Press: Cambridge, 2009.

[ref91] Shah D.; Mjalli F. S. Effect of water on the thermo-physical properties of Reline: An experimental and molecular simulation based approach. Phys. Chem. Chem. Phys. 2014, 16, 23900–23907. 10.1039/C4CP02600D. 25277220

[ref92] Abbott A. P.; Capper G.; Gray S. Design of Improved Deep Eutectic Solvents Using Hole Theory. ChemPhysChem 2006, 7, 803–806. 10.1002/cphc.200500489. 16596609

[ref93] Tu K.-M.; Ishizuka R.; Matubayasi N. Spatial-decomposition analysis of electrical conductivity in concentrated electrolyte solution. J. Chem. Phys. 2014, 141, 044126 10.1063/1.4890741. 25084900

[ref94] Haghbakhsh R.; Raeissi S. Investigation of solutions of ethyl alcohol and the deep eutectic solvent of Reline for their volumetric properties. Fluid Phase Equilib. 2018, 472, 39–47. 10.1016/j.fluid.2018.05.008.

