# Supplementary material for: Solubilities and Transport Properties of CO2, Oxalic Acid, and Formic Acid in Mixed Solvents Composed of Deep Eutectic Solvents, Methanol, and Propylene Carbonate
Source: J Phys Chem B. 2022 May 4;126(19):3572–84. doi: 10.1021/acs.jpcb.2c01425 (PMC9125562; doi:10.1021/acs.jpcb.2c01425)
Supplement: Supplementary file 1 — jp2c01425_si_001.pdf [file jp2c01425_si_001.pdf]

Supporting Information: Solubilities and Transport Properties of CO_2, Oxalic Acid, and Formic Acid in Mixed Solvents Composed of Deep Eutectic Solvents, Methanol, and Propylene Carbonate

Noura Dawass[$],[†] Jilles Langeveld[$],[‡] Mahinder Ramdin,[‡] Elena Pérez-Gallent,[¶] Angel A. Villanueva,[¶] Erwin J. M. Giling,[¶] Jort Langerak,[§] Leo J. P. van den Broeke,[‡] Thijs J. H. Vlugt,[‡] and Othonas A. Moultos[*,‡]

†*Chemical Engineering Program, Texas A&M University at Qatar, P.O. Box 23874, Doha, Qatar*

‡*Engineering Thermodynamics, Process & Energy Department, Faculty of Mechanical, Maritime and Materials Engineering, Delft University of Technology, Leeghwaterstraat 39, 2628CB, Delft, The Netherlands*

¶*Department of Sustainable Process and Energy Systems, TNO, Delft, Zuid-Holland 2628CA, The Netherlands*

§*Research and Development Department, DMT Environmental Technology, Yndustrywei 3, 8501SN, Joure, The Netherlands*

E-mail: o.moultos@tudelft.nl

[$]These authors contributed equally to this work.

In this Supporting Information we provide:

- Force field parameters of all species simulated in this work, i.e., reline, ethaline, propylene carbonate, methanol, oxalic acid, formic acid and carbon dioxide.
- Raw data for the computed densities, transport properties (i.e., viscosities, self–diffusion coefficients, and ionic conductivities), and number of hydrogen bonds (HBs) for the mixtures methanol–reline, methanol–ethaline, and propylene carbonate–ethaline at 298.15 K, 1 bar and mole fractions of DES ranging from 0 to 1.

1 Force fields

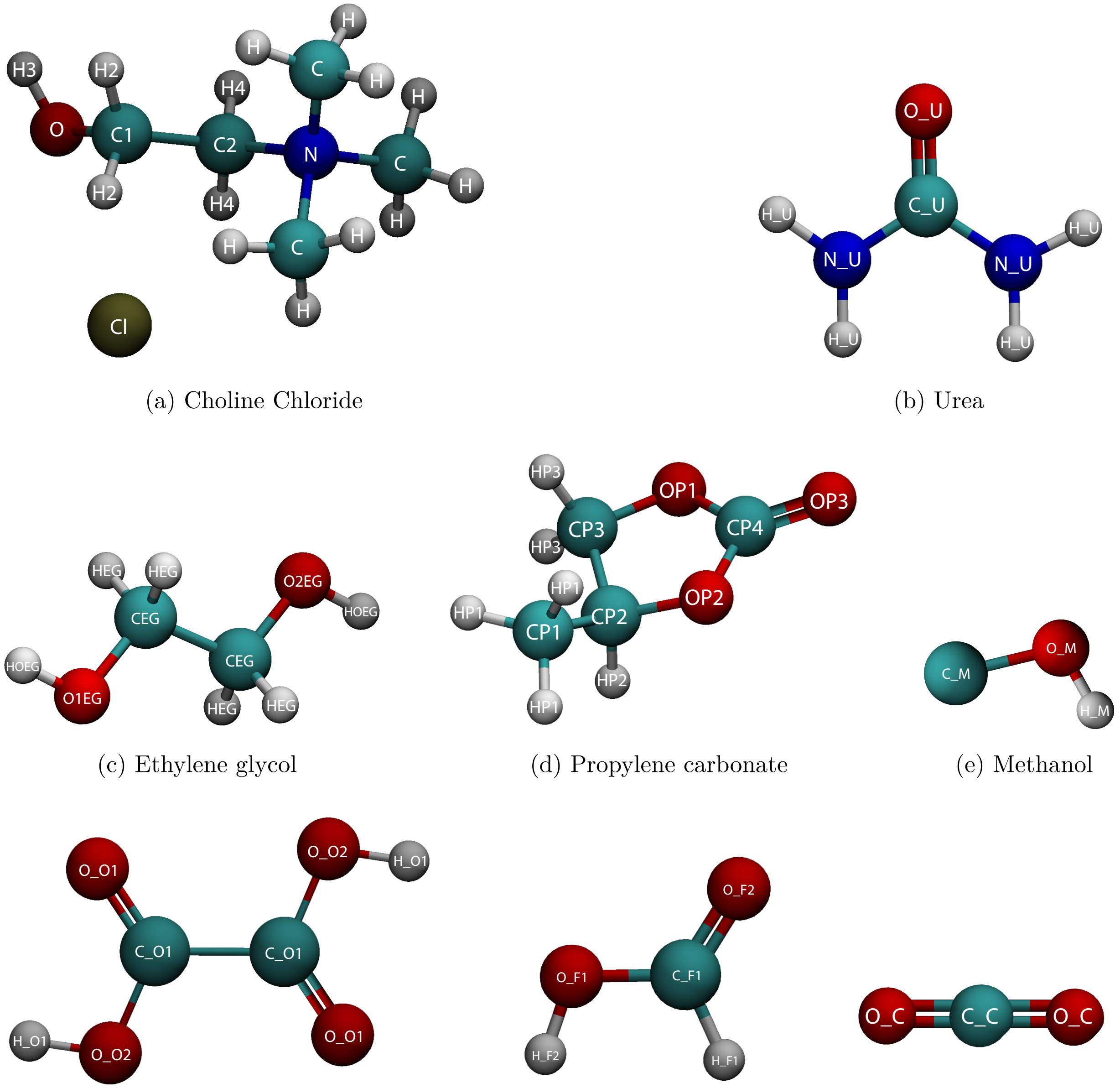

(a) Choline Chloride (b) Urea

(c) Ethylene glycol (d) Propylene carbonate (e) Methanol

(f) Oxalic acid (g) Formic acid (h) Carbon dioxide

Figure S1: Molecular structures and atom labels of the species studied.

Table S1: Non-bonded force field parameters for choline (Ch^+), chloride (Cl^-), ethylene glycol (EG), formic (FA) and oxalic (OA) acid, methanol (MeOH), and propylene carbonate (PC). The atom types are defined in Figure S1 The Lennard-Jones (LJ) energy is calculated as: $U_{\mathrm{LJ}}(r) = 4\epsilon[(\sigma/r)^{12} - (\sigma/r)^6]$, where r is the site-site distance. The electrostatic (Coulomb) energy is calculated as: $U_{\mathrm{Coulomb}}(r) = (q_i q_j)/(4\pi\epsilon_0 r)$, where, q_i and q_j are the charges on sites i and j, and ϵ_0 is the dielectric constant of the vacuum. The charges of Ch^+ and Cl^- shown in the table are scaled by a factor of 0.8 and 0.9 for reline and ethaline, respectively.

Species	Atom type	ϵ/[kcal mol^{-1}]	σ/[Å]	q/[e]	Force field
Ch^+_{reline}	C	0.1094	3.3996	-0.10736	GAFF[1]
	C1	0.1094	3.3996	0.12008	
	C2	0.1094	3.3996	-0.02576	
	H	0.0157	1.9599	0.09544	
	H2	0.0157	2.4713	0.0408	
	H3	0.001	0.1	0.3636	
	H4	0.0157	1.9599	0.08928	
	N	0.17	3.2499	0.04016	
	O	0.2104	3.0664	-0.49512	
Ch^+_{ethaline}	C	0.1094	3.3996	-0.12078	GAFF[1]
	C1	0.1094	3.3996	0.13509	
	C2	0.1094	3.3996	-0.02898	
	H	0.0157	1.9599	0.10737	
	H2	0.0157	2.4713	0.0459	
	H3	0.001	0.1	0.40905	
	H4	0.0157	1.9599	0.10044	
	N	0.17	3.2499	0.04518	
	O	0.2104	3.0664	-0.55701	
Cl^-_{reline}	Cl	0.1	4.401	-0.8	GAFF[1]
Cl^-_{ethaline}	Cl	0.1	4.401	-0.9	GAFF[1]
Urea	C_U	0.086	3.3996	1.0401	GAFF[1]
	H_U	0.0157	1.069	0.4167	
	N_U	0.17	3.25	-1.0246	
	O_U	0.21	2.96	-0.6577	

Continued on next page

Species	Atom type	ϵ/[kcal mol^{-1}]	σ/[Å]	q/[e]	Force field
EG	CEG	0.1094	3.3996	0.1615	GAFF[1]
	HEG	0.0157	2.4713	0.0328	
	HOEG	0.001	0.1	0.4069	
	O1EG	0.2104	3.0664	-0.634	
	O2EG	0.2104	3.0664	-0.634	
CO_2	C_C	0.05365	2.8	0.7	TraPPE[2]
	O_C	0.15698	3.028	-0.35	
FA	C_F1	0.0987	3.67	0.52	modified OPLS[3]
	H_F1	0.0141	2.37	0	
	H_F2	0.002	1	0.45	
	O_F1	0.1599	2.94	-0.53	
	O_F2	0.1974	2.9	-0.44	
OA	C_O1	0.1575	3.75	0.416	modified OPLS[4]
	H_O1	0.001	0.1	0.33	
	O_O1	0.315	2.96	-0.352	
	O_O2	0.255	2.92	-0.394	
MeOH	C_M	0.19475	3.75	0.265	TraPPE[5]
	H_M	0	0	0.435	
	O_M	0.18481	3.02	-0.7	
PC	CP1	0.066	3.5	-0.365	modified GAFF[6]
	CP2	0.066	3.5	0.349	
	CP3	0.066	3.5	0.194	
	CP4	0.105	3.75	0.878	
	HP1	0.015	2.42	0.113	
	HP2	0.015	2.42	0.032	
	HP3	0.015	2.42	0.008	
	OP1	0.17	3	-0.46	
	OP2	0.17	3	-0.507	
	OP3	0.21	2.96	-0.476	

Table S2: Bond-stretching interaction parameters for all species studied (see Table S1 for the force fields used and abbreviations, and Figure S1 for the atom types). The bond-stretching energy is calculated according to: $U_{\text{stretching}}(l) = k_l(l - l_0)^2$, where k_l is the force constant, l and l_0 are the bond length and the equilibrium bond length, respectively.

Species	Bond type	k_l/[kcal mol^{-1}Å^{-2}]	r_0/[Å]
Ch^+	C-H	338.7	1.091
	C-N	293.6	1.499
	C1-C2	303.1	1.535
	C1-H2	335.9	1.093
	C1-O	314.1	1.426
	C2-H4	338.7	1.091
	C2-N	293.6	1.499
	H3-O	369.6	0.974
Urea	C_U-N_U	478.2	1.35
	C_U-O_U	648	1.21
	H_U-N_U	410.2	1.01
EG	CEG-CEG	303.1	1.535
	CEG-HEG	335.9	1.093
	CEG-O1EG	314.1	1.426
	CEG-O2EG	314.1	1.426
	HOEG-O1EG	369.6	0.974
	HOEG-O2EG	369.6	0.974
CO_2	C_C-O_C	2058	1.16
FA	C_F1-H_F1	361.8	1.105
	C_F1-O_F1	383.1	1.351
	C_F1-O_F2	652.6	1.218
	H_F2-O_F1	563.5	0.973
OA	C_O1-C_O1	350	1.51
	C_O1-O_O1	570	1.229
	C_O1-O_O2	450	1.364
	H_O1-O_O2	553	0.945

Continued on next page

Species	Bond type	k_l/[kcal mol^{-1}Å^{-2}]	r_0/[Å]
MeOH	C_M-O_M	1000	1.43
	H_M-O_M	1000	0.945
PC	CP1-CP2	232.5	1.538
	CP1-HP1	375.9	1.097
	CP2-CP3	232.5	1.538
	CP2-HP2	375.9	1.097
	CP2-OP2	284.8	1.432
	CP3-HP3	375.9	1.097
	CP3-OP1	284.8	1.432
	CP4-OP1	372.9	1.358
	CP4-OP2	372.9	1.358
	CP4-OP3	652.6	1.218

Table S3: Bond-bending interaction parameters for all species studied (see Table S1 for the force fields used and abbreviations, and Figure S1 for the atom types). The bond-bending energy is calculated according to: $U_{\text{bending}}(\theta) = k_\theta(\theta - \theta_0)^2$, where k_θ is the force constant, θ and θ_0 are the angle and the equilibrium angle, respectively.

Species	Angle	k_θ/[kcal mol^{-1}rad^{-2}]	θ_0/[°]
Ch^+	C-N-C	62.8	110.64
	C-N-C2	62.8	110.64
	C1-C2-H4	46	111.74
	C1-C2-N	66	108.93
	C1-O-H3	47.1	108.16
	C2-C1-H2	46.4	110.07
	C2-C1-O	67.7	109.43
	H-C-H	39	110.74
	H-C-N	49	107.91
	H2-C1-H2	39.2	109.55
	H2-C1-O	51	109.88
	H4-C2-H4	39	110.74
	H4-C2-N	49	107.91
Urea	C_U-N_U-H_U	49.21	118.5
	H_U-N_U-H_U	39.73	117.9
	N_U-C_U-N_U	74.8	113.4
	N_U-C_U-O_U	75.83	122
EG	CEG-CEG-HEG	46.4	110.07
	CEG-CEG-O1EG	67.7	109.43
	CEG-CEG-O2EG	67.7	109.43
	CEG-O1EG-HOEG	47.1	108.16
	CEG-O2EG-HOEG	47.1	108.16
	HEG-CEG-HEG	39.2	109.55
	HEG-CEG-O1EG	51	109.88
	HEG-CEG-O2EG	51	109.88
CO_2	O_C-C_C-O_C	112	180
FA	C_F1-O_F1-H_F2	51.6	106.55
	H_F1-C_F1-O_F1	65.3	109.49
	H_F1-C_F1-O_F2	65.9	123.65
	O_F1-C_F1-O_F2	115.7	122.1
OA	C_O1-C_O1-O_O1	80	121.4
	C_O1-C_O1-O_O2	70.96	118.03
	C_O1-O_O2-H_O1	35	113
	O_O1-C_O1-O_O2	80	121
MeOH	C_M-O_M-H_M	55	108.5

Continued on next page

Species	Angle	k_θ/[kcal mol^{-1}rad^{-2}]	θ_0/[°]
PC	CP1-CP2-CP3	64.9	111.51
	CP1-CP2-HP2	46.8	109.8
	CP1-CP2-OP2	85.3	107.97
	CP2-CP1-HP1	46.8	109.8
	CP2-CP3-HP3	46.8	109.8
	CP2-CP3-OP1	85.3	107.97
	CP2-OP2-CP4	66.9	115.98
	CP3-CP2-HP2	46.8	109.8
	CP3-CP2-OP2	85.3	107.97
	CP3-OP1-CP4	66.9	115.98
	HP1-CP1-HP1	39	107.58
	HP2-CP2-OP2	62.7	108.7
	HP3-CP3-HP3	39	107.58
	HP3-CP3-OP1	62.7	108.7
	OP1-CP4-OP2	115.3	111.29
	OP1-CP4-OP3	114.8	123.25
	OP2-CP4-OP3	114.8	123.25

Table S4: CHARMM-type[7] dihedral torsion parameters (see Table S1 for the force fields used and abbreviations, and Figure S1 for the atom types). The torsion energy is calculated as: $U_{\mathrm{CHARMM}} = K_\phi[1 + \cos(n\phi - \delta)]$, where K_ϕ is the dihedral angle force constant and ϕ is the dihedral angle.

Species	Dihedral	K_ϕ/[kcal mol^{-1}]	n	δ/[°]
Ch^+	C1-C2-N-C	0.156	3	0
	H-C-N-C	0.156	3	0
	H-C-N-C2	0.156	3	0
	H2-C1-C2-H4	0.156	3	0
	H2-C1-C2-N	0.156	3	0
	H2-C1-O-H3	0.167	3	0
	H4-C2-N-C	0.156	3	0
	O-C1-C2-H4	0.156	3	0
	O-C1-C2-N	0.156	3	0
Urea	N_U-C_U-N_U-H_U	2.5	2	180
EG	HEG-CEG-CEG-HEG	0.156	3	0
	HEG-CEG-CEG-O1EG	0.25	1	0
	HEG-CEG-CEG-O2EG	0.25	1	0
	HEG-CEG-O1EG-HOEG	0.167	3	0
	HEG-CEG-O2EG-HOEG	0.167	3	0
PC	CP1-CP2-CP3-HP3	0.16	3	0
	CP1-CP2-CP3-OP1	0.156	3	0
	CP1-CP2-OP2-CP4	0.383	3	0
	CP2-CP3-OP1-CP4	0.383	3	0
	CP3-CP2-OP2-CP4	0.383	3	0
	HP1-CP1-CP2-CP3	0.16	3	0
	HP1-CP1-CP2-HP2	0.156	3	0
	HP1-CP1-CP2-OP2	0	3	0
	HP2-CP2-CP3-HP3	0.156	3	0
	HP2-CP2-CP3-OP1	0	3	0
	HP2-CP2-OP2-CP4	0.383	3	0
	HP3-CP3-OP1-CP4	0.383	3	0
	OP1-CP4-OP2-CP2	2.7	2	180
	OP2-CP2-CP3-HP3	0	3	0
	OP2-CP2-CP3-OP1	0.144	3	0
	OP2-CP4-OP1-CP3	2.7	2	180
	OP3-CP4-OP1-CP3	2.7	2	180
	OP3-CP4-OP2-CP2	2.7	2	180

Table S5: OPLS-type[8] dihedral torsion parameters (see Table S1 for the force fields used and abbreviations, and Figure S1 for the atom types). The torsion energy is calculated as: $U_{\mathrm{OPLS}} = \frac{1}{2}K_1[1+\cos(\phi)] + \frac{1}{2}K_2[1+\cos(2\phi)] + \frac{1}{2}K_3[1+\cos(3\phi)]$, where K_1, K_2 and K_3 are the dihedral angle force constants and ϕ is the dihedral angle.

Species	Dihedral	K_1/[kcal mol^{-1}]	K_2/[kcal mol^{-1}]	K_3/[kcal mol^{-1}]
Ch^+	C2-C1-O-H3	0.5	0	0.32
Urea	O_U-C_U-N_U-H_U	4	5	0
EG	CEG-CEG-O1EG-HOEG	0.5	0	0.32
	CEG-CEG-O2EG-HOEG	0.5	0	0.32
OA	C_O1-C_O1-O_O2-H_O1	3	5.5	0
	O_O1-C_O1-C_O1-O_O1	1.6	3.2	0
	O_O1-C_O1-C_O1-O_O2	1.6	3.2	0
	O_O1-C_O1-O_O2-H_O1	0	5.5	0
	O_O2-C_O1-C_O1-O_O2	1.6	3.2	0

Table S6: Multi/harmonic-type[9] dihedral torsion parameters (see Table S1 for the force fields used and abbreviations, and Figure S1 for the atom types). The torsion energy is calculated as: $U_{\mathrm{multi/harmonic}} = \sum_{n=1}^{4} K_n \cos^{n-1}(\phi)$, where K_n are the dihedral angle force constants and ϕ is the dihedral angle.

Species	Dihedral	K_1, K_2, K_3, K_4/[kcal mol^{-1}]
EG	O1EG-CEG-CEG-O2EG	0.144, -0.432, 2.35, 0.576
FA	H_F1-C_F1-O_F1-H_F2	5.1864, -0.62141, -4.565, 0
	O_F2-C_F1-O_F1-H_F2	4.565, 0, -4.565, 0

Table S7: Improper torsion parameters (CVFF-type[9]) (see Table S1 for the force fields used and abbreviations, and Figure S1 for the atom types). The torsion energy is calculated as: $U_{\mathrm{Improper}} = K[1 + d\cos(n\phi)]$, where K is the dihedral angle force constant and ϕ is the dihedral angle.

Molecule	Improper	K[kcal mol^{-1}]	d	n
Urea	C_U-H_U-N_U-H_U	1.1	-1	2
	N_U-N_U-C_U-O_U	10.5	-1	2
FA	H_F1-O_F1-C_F1-O_F2	1.1	-1	2
OA	C_O1-O_O1-C_O1-O_O2	1.1	-1	2
PC	OP1-OP2-CP4-OP3	1.1	-1	2

2 Results

Table S8: Densities (ρ), shear visosities (η), and ionic conductivities (κ) of the reline-MeOH, ethaline-MeOH, and ethaline-PC mixtures. The numbers in the parentheses are the standard deviations. The computed standard deviations of the densities are in the range of 1-2%.

	ρ/[kg m^{-3}]		
x_{DES}	Reline-MeOH	Ethaline-MeOH	Ethaline-PC
0	778	778	1200
0.1	850	839	1190
0.2	916	886	1190
0.4	1020	971	1170
0.6	1100	1030	1160
0.8	1166	1080	1140
1	1220	1120	1120
	η/[mPa s]		
0	0.50 (0.07)	0.50 (0.07)	3.01 (0.3)
0.1	1.0 (0.1)	0.87 (0.06)	4.0 (0.5)
0.2	1.8 (0.2)	1.5 (0.2)	5.3 (0.8)
0.4	4.7 (0.5)	3.5 (0.4)	8 (1)
0.6	14 (1)	9 (1)	16 (4)
0.8	72 (13)	21 (3)	33 (5)
1	455 (91)	62 (14)	62 (14)
	κ/[S m^{-1}]		
0.1	4.5 (0.1)	3.9 (0.1)	0.44 (0.04)
0.2	5.69 (0.01)	5.4 (0.2)	0.69 (0.04)
0.4	4.4 (0.1)	4.8 (0.2)	0.94 (0.06)
0.6	2.1 (0.1)	3.0 (0.1)	0.94 (0.04)
0.8	0.63 (0.05)	1.5 (0.1)	0.77 (0.04)
1	0.110 (0.008)	0.63 (0.03)	0.63 (0.03)

Table S9: Finite-size corrected self-diffusion coefficients ($D/[10^{-11}\ m^2\ s^{-1}]$) of all species in the reline-MeOH, ethaline-MeOH, and ethaline-PC mixtures (see Table S1 for the abbreviations). The numbers in the parentheses are the standard deviations.

Reline-MeOH

x_{DES}	Ch^+	Cl^+-	Urea	MeOH	CO_2	OA	FA
0	-	-	-	277 (20)	697 (11)	245 (22)	294 (24)
0.1	94 (3)	107 (3)	103 (2)	175 (3)	425 (13)	101 (7)	160 (6)
0.2	58 (2)	70 (1)	64 (2)	121 (2)	282 (14)	66 (4)	104 (6)
0.4	23 (1)	29 (1)	26 (1)	57 (1)	108 (11)	27 (2)	47 (3)
0.6	7.6 (0.5)	9.8 (0.5)	9.4 (0.5)	23 (1)	42 (4)	8.5 (0.8)	18 (2)
0.8	1.9 (0.2)	2.4 (0.2)	2.8 (0.2)	6.9 (0.5)	13 (2)	2.2 (0.2)	6.1 (0.5)
1	0.27 (0.03)	0.35 (0.02)	0.50 (0.05)	-	2.0 (0.1)	0.40 (0.02)	0.90 (0.03)

Ethaline-MeOH

x_{DES}	Ch^+	Cl^+-	EG	MeOH	CO_2	OA	FA
0	-	-	-	277 (20)	697 (11)	245 (22)	294 (24)
0.1	86 (3)	90 (5)	127 (10)	172 (6)	399 (17)	146 (35)	142 (10)
0.2	59 (2)	65 (3)	87 (6)	126 (5)	298 (13)	105 (30)	103 (7)
0.4	27 (1)	32 (2)	42 (3)	65 (3)	131 (3)	50 (17)	49 (2)
0.6	11 (1)	15 (1)	19 (1)	32 (1)	61 (3)	25 (10)	24 (1)
0.8	4.8 (0.2)	6.5 (0.2)	8.6 (0.4)	15.0 (0.8)	24 (2)	11 (4)	12 (2)
1	1.7 (0.1)	2.3 (0.1)	3.0 (0.2)	-	9 (1)	2.6 (0.5)	4.3 (0.5)

Ethaline-PC

x_{DES}	Ch^+	Cl^+-	EG	PC	CO_2	OA	FA
0	-	-	-	41 (2)	114 (9)	39 (2)	61 (5)
0.1	18 (2)	20 (2)	28 (2)	34 (1)	92 (12)	27.3 (0.9)	31 (2)
0.2	15 (1)	15 (1)	22 (1)	28.1 (0.6)	79 (7)	21 (1)	25 (2)
0.4	8.8 (0.7)	9.6 (0.7)	14 (1)	18.7 (0.9)	59 (7)	14 (1)	17.5 (0.9)
0.6	5.3 (0.3)	6.2 (0.4)	9.5 (0.4)	11.2 (0.7)	37 (2)	9 (1)	13 (2)
0.8	2.9 (0.1)	3.7 (0.2)	5.6 (0.3)	5.2 (0.4)	20 (3)	4.0 (0.3)	6.8 (0.6)
1	1.7 (0.1)	2.3 (0.1)	3.2 (0.2)	-	9 (1)	2.6 (0.5)	4.3 (0.5)

Table S10: Hydrogen bonds between all species in the reline-MeOH, ethaline-MeOH, and ethaline-PC mixtures (see Table S1 for the abbreviations). The computed numbers of hydrogen bonds are normalized to represent a system of 100 DES molecules. The number of molecules of the organic solvent follows from the mole fraction of the DES. The numbers in the parentheses are the standard deviations.

Reline-MeOH					
x_{DES}	Cl^--Ch^+	Cl^--urea	Ch^+-Ch^+	Ch^+-rea	urea-urea
0.1	3 (1)	9.05 (3)	0.05 (0.05)	4 (1)	25 (5)
0.2	5 (1)	16 (3)	0.1 (0.1)	7 (2)	41 (6)
0.4	8 (2)	29 (4)	0.2 (0.2)	11 (2)	62 (7)
0.6	10 (1)	39 (4)	0.3 (0.3)	15 (2)	78 (7)
0.8	12 (2)	49 (4)	0.3 (0.3)	17 (2)	90 (7)
1	15 (2)	59 (3)	0.4 (0.4)	20 (2)	101 (6)
Ethaline-MeOH					
x_{DES}	Cl^--Ch^+	Cl^--EG	Ch^+-Ch^+	Ch^+-EG	EG-EG
0.1	5 (1)	14 (3)	0.05 (0.05)	3 (1)	10 (3)
0.2	9 (2)	25 (3)	0.1 (0.1)	5 (2)	16 (4)
0.4	14 (3)	44 (6)	0.1 (0.1)	8 (2)	26 (5)
0.6	18 (3)	60 (7)	0.2 (0.2)	10 (2)	33 (6)
0.8	23 (3)	74 (8)	0.2 (0.2)	11 (2)	38 (6)
1	27 (2)	86 (3)	0.3 (0.3)	13 (2)	42 (4)
Ethaline-PC					
x_{DES}	Cl^--Ch^+	Cl^--EG	Ch^+-Ch^+	Ch^+-EG	EG-EG
0.1	24 (5)	85 (11)	0.1 (0.1)	7 (4)	13 (8)
0.2	26 (4)	88 (7)	0.2 (0.2)	8 (3)	19 (6)
0.4	27 (3)	88 (5)	0.2 (0.2)	10 (2)	27 (6)
0.6	27 (2)	87 (4)	0.2 (0.2)	11 (2)	32 (5)
0.8	27 (2)	87 (4)	0.2 (0.2)	12 (2)	37 (5)
1	27 (2)	86 (3)	0.3 (0.3)	13 (2)	42 (4)

Table S11: Hydrogen bonds between MeOH and other species in the reline-MeOH and ethaline-MeOH mixtures (see Table S1 for the abbreviations). The computed numbers of hydrogen bonds are normalized to represent a system of 100 MeOH molecules. The number of DES molecules follows from the mole fraction of the DES. The numbers in the parentheses are the standard deviations.

Reline-MeOH

x_{DES}	Meth-Cl^-	Meth-Ch^+	Meth-Urea	Meth-Meth
0.0	-	-	-	77 (4)
0.1	5.4 (0.3)	1.8 (0.1)	15 (1)	69 (2)
0.2	10.0 (0.6)	3.3 (0.3)	27 (1)	57 (2)
0.4	18 (1)	5.9 (0.8)	46 (3)	38 (3)
0.6	25 (2)	8 (1)	63 (4)	23 (4)
0.8	31 (4)	10 (2)	78 (6)	11 (4)

Ethaline-MeOH

x_{DES}	Meth-Cl^-	Meth-Ch^+	Meth-EG	Meth-Meth
0	-	-	-	77 (4)
0.1	7.2 (0.3)	2.1 (0.2)	11.4 (0.6)	67 (2)
0.2	13.6 (0.9)	3.8 (0.4)	20 (2)	54 (3)
0.4	25 (3)	7 (1)	34 (4)	34 (4)
0.6	34 (4)	9 (2)	44 (5)	20 (4)
0.8	42 (5)	11 (3)	52 (7)	9 (3)

Table S12: Hydrogen bonds between PC and other species in the ethaline-PC mixture (see Table S1 for the abbreviations). The computed numbers of hydrogen bonds are normalized to represent a system of 100 PC molecules. The number of ethaline molecules follows from the mole fraction of the DES. The numbers in the parentheses are the standard deviations.

Ethaline-PC		
x_{DES}	PC-Ch^+	PC-EG
0.1	0.4 (0.2)	1.2 (0.4)
0.2	0.5 (0.2)	1.9 (0.5)
0.4	0.6 (0.4)	3.2 (0.9)
0.6	0.7 (0.4)	5 (1)
0.8	0.8 (0.7)	6 (2)

References

(1) Wang, J.; Wolf, R. M.; Caldwell, J. W.; Kollman, P. A.; Case, D. A. Development and Testing of a General Amber Force Field. *J. Comput. Chem.* **2004**, *25*, 1157–1174.

(2) Potoff, J. J.; Siepmann, J. I. Vapor–liquid equilibria of mixtures containing alkanes, carbon dioxide, and nitrogen. *AIChE J.* **2001**, *47*, 1676–1682.

(3) Salas, F. J.; Núñez-Rojas, E.; Alejandre, J. Stability of formic acid/pyridine and isonicotinamide/formamide cocrystals by molecular dynamics simulations. *Theor. Chem. Acc.* **2017**, *136*, 1–12.

(4) Doherty, B.; Acevedo, O. OPLS Force Field for Choline Chloride-Based Deep Eutectic Solvents. *J. Phys. Chem. B* **2018**, *122*, 9982–9993.

(5) Chen, B.; Potoff, J. J.; Siepmann, J. I. Monte Carlo calculations for alcohols and their mixtures with alkanes. Transferable potentials for phase equilibria. 5. United–atom description of primary, secondary, and tertiary alcohols. *J. Phys. Chem. B* **2001**, *105*, 3093–3104.

(6) Silva, L. B.; Freitas, L. C. G. Structural and thermodynamic properties of liquid ethylene carbonate and propylene carbonate by Monte Carlo Simulations. *J. Mol. Struct.* **2007**, *806*, 23–34.

(7) MacKerell, A. D. et al. All-Atom Empirical Potential for Molecular Modeling and Dynamics Studies of Proteins. *J. Phys. Chem. B* **1998**, *102*, 3586–3616.

(8) Sun, H.; Li, Y.; Wu, X.; Li, G. Theoretical study on the structures and properties of mixtures of urea and choline chloride. *J. Mol. Model.* **2013**, *19*, 2433–2441.

(9) Plimpton, S. Fast Parallel Algorithms for Short-Range Molecular Dynamics. *J. Comput. Phys.* **1995**, *117*, 1–19.
